# Safety and effectiveness of apixaban versus warfarin for acute venous thromboembolism in patients with end‐stage kidney disease: A national cohort study

**DOI:** 10.1002/jhm.12926

**Published:** 2022-08-05

**Authors:** Michael I. Ellenbogen, Shirin Ardeshirrouhanifard, Jodi B. Segal, Michael B. Streiff, Steven B. Deitelzweig, Daniel J. Brotman

**Affiliations:** ^1^ Department of Medicine Johns Hopkins School of Medicine Baltimore Maryland USA; ^2^ Hopkins Business of Health Initiative Johns Hopkins University Baltimore Maryland USA; ^3^ Department of Epidemiology Johns Hopkins Bloomberg School of Public Health Baltimore Maryland USA; ^4^ Department of Health Policy and Management, and Epidemiology Johns Hopkins Bloomberg School of Public Health Baltimore Maryland USA; ^5^ Departments of Medicine and Pathology Johns Hopkins School of Medicine Baltimore Maryland USA; ^6^ Department of Medicine Ochsner Health System New Orleans Louisiana USA; ^7^ Department of Medicine Johns Hopkins School of Medicine Baltimore Maryland USA

## Abstract

**Background:**

Patients with end‐stage kidney disease (ESKD) are at significantly increased risk for both thrombosis and bleeding relative to those with normal renal function. The optimal therapy of venous thromboembolism (VTE) in patients with ESKD is unknown.

**Objective:**

To compare the safety and effectiveness of apixaban relative to warfarin in patients with ESKD and acute VTE.

**Design, Setting and Participants:**

New‐user, active‐comparator retrospective United States population‐based cohort with inverse probability of treatment weighting, using the United States Renal Data System data from 2014 to 2018. We included adults with ESKD on hemodialysis or peritoneal dialysis who were newly initiated on apixaban or warfarin for an acute VTE.

**Main Outcome and Measures:**

The coprimary outcomes were major bleeding, recurrent VTE, and all‐cause mortality within 6 months of anticoagulant initiation. Secondary outcomes were intracranial hemorrhage and gastrointestinal bleeding. The primary analyses were based on intent‐to‐treat defined by the first drug received and accounted for competing risks of death. Sensitivity analyses included varied follow‐up time, as‐treated analyses, and dose‐specific apixaban subgroups.

**Results:**

The apixaban and warfarin cohorts included 2302 and 9263 patients, respectively. Apixaban was associated with a lower risk of major bleeding (hazard ratio [HR] 0.81, 95% confidence interval [CI]: 0.70–0.94), intracranial bleeding (HR 0.69, 95% CI 0.48–0.98), and gastrointestinal bleeding (HR 0.82, 95% CI 0.69–0.96). Recurrent VTE and all‐cause mortality were not significantly different between the groups.

**Conclusion:**

Apixaban was associated with a lower risk of bleeding relative to warfarin when used to treat acute VTE in patients with ESKD on dialysis.

## INTRODUCTION

Patients with end‐stage kidney disease (ESKD) are at significantly increased risk for both thrombosis and bleeding relative to those with normal renal function, which makes anticoagulation particularly challenging.^1^, ^2^, ^3^ Furthermore, patients with ESKD initiating anticoagulation for venous thromboembolism (VTE) are typically hospitalized to receive parenteral heparin when starting warfarin, putting them at risk for hospital‐associated complications, including heparin‐induced thrombocytopenia. A safe, effective therapy for this high‐risk population is needed.

The direct oral anticoagulants (DOACs)—apixaban, dabigatran, edoxaban, and rivaroxaban—have provided options for the management of VTE but have not been extensively evaluated in individuals with ESKD for this indication. Rivaroxaban and dabigatran have been associated with a higher risk of bleeding than warfarin in patients with ESKD who are anticoagulated for atrial fibrillation (AF) in observational studies.^4^, ^5^ A small randomized study of reduced dose rivaroxaban (10 mg daily) versus warfarin noted similar rates of major bleeding, stroke, and systemic embolism in this population although it was underpowered to demonstrate noninferiority for these clinically important outcomes.^6^ Apixaban may be safer than warfarin^7^, ^8^, ^9^ with recent evidence supporting lower bleeding rates in patients with ESKD treated for AF.^10^, ^11^ RENAL‐AF, an open randomized controlled trial (RCT) of apixaban versus warfarin for atrial fibrillation in patients on hemodialysis was halted prematurely due to slow accrual. Therefore, high‐quality RCT data evaluating DOACs for patients with ESKD and AF are lacking. Similarly, the safety and effectiveness of apixaban relative to warfarin in patients with ESKD and acute VTE have not been extensively studied. There is reason to speculate that bleeding risk and risk of recurrent thrombosis could be different in patients with AF compared to VTE treated with apixaban. Patients with VTE are generally treated with higher doses of apixaban (10 mg twice daily for 1 week followed by 5 mg twice daily); in AF, there is no role for doses greater than 5 mg twice daily at treatment initiation, and some patients can be treated with 2.5 mg twice daily, depending on patient factors.^12^ In contrast, in VTE, the use of less than 5 mg twice daily—regardless of patient characteristics—is off‐label. We sought to characterize recent trends in the use of apixaban to treat VTE in ESKD and evaluate the safety and effectiveness of apixaban compared to warfarin. We hypothesized a lower bleeding risk and similar effectiveness of apixaban when compared to warfarin.

## METHODS

### Data source, study design, and population

The United States Renal Data System (USRDS) is a national data system that collects, analyses, and distributes information about chronic kidney disease (CKD) and ESKD in the United States.^13^ It includes all available Medicare Parts A, B, and D claims data from affected individuals and supplements this with extensive data provided by dialysis centers. The USRDS includes information about these Medicare beneficiaries' healthcare service use including inpatient and outpatient encounters and outpatient prescription medication use. We used institutional claims, physician/supplier (carrier), and Medicare part D claims from 2006 to 2018 and MedPAR (inpatient) and outpatient claims from 2011 to 2018 from the USRDS. This study received approval from the Johns Hopkins School of Medicine Institutional Review Board and the protocol was posted on clinicaltrials.gov (NCT04818151) upon initiation.

We performed a new‐user, active‐comparator retrospective cohort study. Patients aged 18 years or greater with ESKD on dialysis, and fee‐for‐service Medicare as their primary insurance, who were newly initiated on either apixaban or warfarin between January 1, 2014 and June 30, 2018, were eligible for inclusion. We required 6 months of continuous Medicare enrollment in Parts A, B, and D before initiation of anticoagulation for inclusion. For cohort entry, we required a diagnosis of VTE using the International Classification of Disease (ICD)‐9 and ICD‐10 codes (Supporting Information: Appendix Table 1) from 30 days before to 7 days after the initial prescription for apixaban or warfarin. We excluded patients with a claim indicating AF or who were admitted to hospice within the 6 months prior to anticoagulant initiation. Additionally, we excluded patients with any anticoagulant use (DOAC or warfarin) in the 30 days prior to cohort entry or 60 or more cumulative days of anticoagulant treatment (DOAC, warfarin, unfractionated heparin, or low‐molecular‐weight heparin) for any diagnosis in the 6 months prior to cohort entry (Supporting Information: Appendix Figure 1).

We stratified the population by treatment and characterized the cohorts by age (continuous variable), gender (binary variable), race (Black, White, other), ethnicity (Hispanic, non‐Hispanic), eligible for Medicaid (yes/no), calendar year of index medication fill, and duration on dialysis (categorical variable). We tabulated the prevalence of comorbid conditions that are most prevalent in this population and/or associated with thrombosis or bleeding. These comorbidities were identified using ICD‐9 and 10, Healthcare Common Procedure Coding System, Current Procedural Terminology, and Diagnosis Related Group codes as well as USRDS‐indicator variables. Where available, we used algorithms from the Centers for Medicare and Medicaid Services (CMS) Chronic Conditions Warehouse^14^ and prior literature^15^, ^16^, ^17^, ^18^ to define these comorbidities (Supporting Information: Appendix Table 2). We also extracted information on concurrent medication use, focusing on medications associated with bleeding and thrombosis.

### Outcomes and follow‐up

All outcomes were prespecified. A primary outcome was major bleeding within 6 months of anticoagulant initiation, defined as either (a) bleeding associated with death, (b) critical site bleeding requiring hospitalization, or (c) bleeding at any site requiring hospitalization with transfusion.^19^ We further classified these major bleeding events as fatal or nonfatal. Secondary bleeding outcomes were clinically relevant nonmajor bleeding (defined as critical site bleeds not requiring hospitalizations and noncritical site bleeds requiring hospitalizations but not transfusions), intracranial bleeding, gastrointestinal (GI) bleeding, and the number of transfusion events per patient (Supporting Information: Appendix Table 3). The co‐primary outcomes were recurrent VTE and all‐cause mortality within 6 months of anticoagulant initiation. Recurrent VTE required a new diagnosis code of VTE in the primary position during a hospitalization beginning more than 21 days after the index anticoagulant prescription.

Follow‐up continued until the earliest of disenrollment from Medicare (parts A, B, or D), kidney transplantation, enrollment in hospice, death, or 6 months. A patient was able to contribute multiple clinical outcomes (clinically relevant nonmajor, nonfatal major, and fatal major bleed, recurrent VTE). We counted only a single bleeding event (i.e., the most severe event) and/or thrombotic event for a patient during a given hospitalization.

### Statistical analyses

We used descriptive statistics to characterize the study population. We calculated the percentage of patients with each of the primary outcomes by treatment group. Additionally, we characterized the proportion of the apixaban group that was prescribed the 5 mg tablet strength exclusively, the 2.5 mg (off‐label) tablet strength exclusively, or a combination of these two strengths. We calculated the mean daily dose per patient for apixaban users.

The main analyses were based on the intent‐to‐treat principle, with exposure defined by the medication first prescribed. We generated a propensity score by modeling the probability of treatment with apixaban rather than warfarin for each cohort member using a logistic regression model including all clinical covariates described above. We then weighted the individuals in the cohort using inverse probability of treatment weights (IPTW) after truncating the weights at the 1st and 99th percentiles. The standardized mean difference between the two cohorts is reported for the unweighted and weighted study populations.

The main analysis used the IPTW‐weighted population and estimated a subdistribution hazard, and 95% confidence interval (CI), for each outcome, with death considered as a competing risk for bleeding and recurrent VTE outcomes. The hazard ratio (HR) for death was generated with a Cox proportional hazard model. We included an indicator of treatment initiation year in the models to adjust for changing use patterns over time and other secular trends. To compare transfusion events between the two cohorts, we used Poisson regression by applying IPTW and adjusting for treatment initiation year as well as the length of follow‐up.

We generated cumulative incidence and survival curves based on the cumulative incidence functions of major bleeding, clinically relevant nonmajor bleeding, intracranial bleeding, gastrointestinal bleeding, recurrent VTE, and all‐cause mortality with direct adjustment (rather than IPTW) for all covariates and adjustment for index year.^20^, ^21^, ^22^

We conducted secondary analyses, varying the follow‐up time to 3 months and 1 month to learn if the treatment effects varied early in treatment. We also conducted sensitivity analyses using an as‐treated rather than intent‐to‐treat methodology. For the as‐treated analysis, discontinuation of the initial treatment (with or without switching to another anticoagulant) defined as more than 30 days without the index medication were censoring events in addition to those included in the intent‐to‐treat analysis.

We conducted exploratory subgroup analyses using an intent‐to‐treat methodology in which we compared outcomes between the exclusively 2.5 mg and exclusively 5 mg apixaban subgroups, the 2.5 mg apixaban subgroup and warfarin, and the 5 mg apixaban subgroup and warfarin. These subgroup analyses used direct adjustment for comorbidities and concurrent medications, rather than IPTW. We also performed a sensitivity analysis comparing the outcomes between the entire apixaban cohort and the warfarin cohort using direct adjustment rather than IPTW to control for clinical characteristics and concurrent medications.

Analyses were conducted using SAS, version 9.4.

## RESULTS

The apixaban and warfarin cohorts included 2302 and 9263 patients, respectively (Supporting Information: Appendix Table 4). The mean age of individuals in the apixaban and warfarin cohorts was 59.8 years (standard deviation [SD] 15.1 years) and 58.3 (SD 15.2). The apixaban and warfarin cohorts were 45.9% and 44.6% White, respectively, and were 55.3% and 54.1% female (Table 1). The dialysis modality at the index date was hemodialysis for 93.8% and peritoneal dialysis for 6.2%. The groups were similar even prior to weighting and were very well‐matched after weighting (Table 2). The proportion of apixaban users in the study population increased markedly during the study period from 2% in 2014 to 47% in 2018 (Supporting Information: Appendix Figure 2). Half of the patients in the apixaban cohort filled prescriptions exclusively for 5 mg tablets (50.0%), while 40.5% exclusively filled 2.5 mg tablets and 9.5% filled a combination of the two tablet strengths. The mean daily dose per patient for apixaban users was 6.7 mg (SD 3.0 mg).

**Table 1:** Patient demographics

	Unweighted study population			Weighted study population		
Characteristic	Apixaban	Warfarin	Standardized mean difference	Apixaban	Warfarin	Standardized mean difference
Number	2302	9263				
Age at index date, years						
Mean (SD)	59.8 (15.1)	58.3 (15.2)	0.098	58.7 (15.3)	58.6 (15.1)	0.008
Gender (%)						
Male	1029 (44.7)	4253 (45.9)		45.7	45.7	
Female	1273 (55.3)	5010 (54.1)	0.024	54.3	54.3	0.000
Race (%)						
White	1057 (45.9)	4127 (44.6)	−0.027	45.1	44.9	0.006
Black/African American	1158 (50.3)	4785 (51.7)	0.027	51.1	51.4	−0.006
Other	87 (3.8)	351 (3.8)	0.0005	3.8	3.8	0.001
Ethnicity (%)						
Hispanic	312 (13.6)	1089 (11.8)	−0.054	12.3	12.1	0.005
Non‐Hispanic	1987 (86.3)	8156 (88.1)	0.052	87.5	87.7	−0.005
Unknown	3 (0.1)	18 (0.2)	0.016	0.2	0.2	0.001
Dual‐eligible (%)	989 (43.0)	4449 (48.0)	0.102	46.5	47.0	−0.01
Year of index medication fill (%)			Not included in propensity score	Not included in propensity score	Not included in propensity score	Not included in propensity score
2014	46 (2.0)	2688 (29.0)				
2015	282 (12.3)	2144 (23.2)				
2016	611 (26.5)	2042 (22.0)				
2017	771 (33.5)	1728 (18.7)				
2018	592 (25.7)	661 (7.1)				
Time on dialysis (years) (%)						
<1	156 (6.8)	718 (7.8)	0.038	7.5	7.6	−0.003
1–2	335 (14.6)	1357 (14.7)	0.0028	14.8	14.6	0.004
2–3	261 (11.3)	1117 (12.1)	0.022	11.8	11.9	−0.002
≥3	1538 (66.8)	5991 (64.7)	−0.045	65.2	65.1	0.002
Unknown	12 (0.5)	80 (0.9)	0.041	0.7	0.8	−0.01
Dialysis modality at index date						
Hemodialysis	2183 (94.8)	8685 (93.8)		94.0	94.0	
Peritoneal dialysis	119 (5.2)	578 (6.2)	0.046	6.0	6.0	−0.002

**Table 2:** Comorbidities and medication exposures at baseline

	Unweighted study population			Weighted study population		
Comorbidity or medication	Apixaban (*N* = 2302)	Warfarin (*N* = 9263)	Standardized mean difference	Apixaban	Warfarin	Standardized mean difference
Anemia (%)	2295 (99.7)	9241 (99.8)	0.013	99.7	99.8	−0.005
Diabetes (%)	1592 (69.2)	6137 (66.3)	−0.062	67.1	66.8	0.005
Hypertension (%)	2240 (97.3)	9027 (97.5)	0.009	97.4	97.4	−0.002
Ischemic heart disease (%)	1268 (55.1)	5021 (54.2)	−0.018	54.4	54.4	0.0007
Obesity (%)	1366 (59.3)	5172 (55.8)	−0.071	56.6	56.5	0.002
Peptic ulcer disease (%)	109 (4.7)	464 (5.0)	0.013	4.9	5.0	−0.0006
Pulmonary hypertension (%)	192 (8.3)	1042 (11.3)	0.098	10.2	10.7	−0.02
Smoker (%)	728 (31.6)	3100 (33.5)	0.039	32.8	33.1	−0.006
Prior VTE (%)	963 (41.8)	3352 (36.2)	−0.116	38.1	37.3	0.015
Prior GI bleeding (%)	311 (13.5)	1336 (14.4)	0.026	14.2	14.2	−0.002
Peripheral arterial disease (%)	830 (36.1)	3356 (36.2)	0.0036	36.0	36.2	−0.004
Liver disease (except viral hepatitis)	198 (8.6)	843 (9.1)	0.018	9.0	9.0	0.0004
Active malignancy (%)	240 (10.4)	992 (10.7)	0.009	10.6	10.7	−0.002
Prescence of central catheter (%)	1282 (55.7)	5708 (61.6)	0.121	60.0	60.4	−0.009
Recent trauma^a^ (%)	406 (17.6)	1756 (19.0)	0.034	18.4	18.7	−0.007
Recent surgery^b^ (%)	714 (31.0)	3113 (33.6)	0.055	32.7	33.1	−0.007
Heart failure (%)	954 (41.4)	3593 (38.8)	−0.054	39.7	39.3	0.007
Prior major bleeding	226 (9.8)	951 (10.3)	0.015	10.1	10.2	−0.002
Stroke or TIA	381 (16.6)	1531 (16.5)	−0.0006	16.5	16.5	−0.002
Antiplatelet (other than aspirin)^c^ (%)	512 (22.2)	1840 (19.9)	−0.058	20.4	20.3	0.002
Vasodilators^d^ (%)	45 (1.9)	149 (1.6)	−0.026	1.7	1.7	−0.0007
Cox2 selective NSAIDs (%)	15 (0.7)	44 (0.5)	−0.024	0.5	0.5	0.0001
Cox2 nonselective NSAIDs (%)	212 (9.2)	755 (8.2)	−0.038	8.3	8.4	−0.003
PPI (%)	1019 (44.3)	4189 (45.2)	0.019	44.8	45.0	−0.005
Antidepressants^e^ (%)	641 (27.9)	2468 (26.6)	−0.027	27.5	26.9	0.013
H2 blockers (%)	304 (13.2)	1069 (11.5)	−0.051	11.9	11.9	0.002
Continuous use of oral corticosteroids (<1 week)	213 (9.3)	836 (9.0)	−0.0079	8.9	9.1	−0.005
Continuous use of oral corticosteroids (≥1 week)	304 (13.2)	1175 (12.7)	−0.016	13.2	12.8	0.01
Erythropoiesis stimulating agents	2083 (90.5)	8414 (90.8)	0.012	90.7	90.8	−0.001
Aspirin^f^ (%)	13 (0.6)	63 (0.7)	Not included in propensity score	Not included in propensity score	Not included in propensity score	Not included in propensity score

Abbreviations: COX, cyclooxygenase; NSAIDs, nonsteroidal anti‐inflammatory drugs; PPI, proton pump inhibitor; TIA, transient ischemic attack; USRDS,United States Renal Data System; VTE, venous thromboembolism.

^a^ Recent trauma defined as up to 30 days before index date (Supporting Information: Appendix Table 2).

^b^ Recent surgery defined as up to 60 days before index date (Supporting Information: Appendix Table 2).

^c^ Clopidogrel, prasugrel, ticagrelor, ticlodipine.

^d^ Cilostazol, dipyramidole.

^e^ Citalopram, escitalopram, fluoxetine, fluvoxamine, paroxetine, sertraline, nefazodone, trazodone, amitriptyline, clomipramine.

^f^ Prescription use only; USRDS does not include over‐the‐counter medications.

Major bleeding affected 13.0% of the study population, with rates of 10.3% and 13.7% in the apixaban and warfarin groups, respectively. GI bleeding affected 10.0% of the entire cohort, with rates of 8.6% and 10.4% in the apixaban and warfarin groups (Table 3).

**Table 3:** Bleeding and thrombotic outcomes at 6 months

	Total	Apixaban	Warfarin
Number of patients, *N*	11,565	2302	9263
Total major bleeding^a^			
Patients, *N* (%)	1507 (13.0)	238 (10.3)	1269 (13.7)
IPTW and index year adjusted, HR (95% CI)		**0.81 (0.70**–**0.94)**	Ref.
Fatal major bleeding			
Patients, *N* (%)	297 (2.6)	44 (1.9)	253 (2.7)
IPTW and index year adjusted, HR (95% CI)		0.71 (0.51–1.00)	Ref.
Nonfatal major bleeding			
Patients, *N* (%)	1239 (10.7)	199 (8.6)	1040 (11.2)
IPTW and index year adjusted, HR (95% CI)		0.85 (0.72–1.004)	Ref.
Total clinically relevant nonmajor bleeding			
Patients, *N* (%)	2026 (17.5)	351 (15.3)	1675 (18.1)
IPTW and index year adjusted, HR (95% CI)		**0.84 (0.74**–**0.94)**	Ref.
Intracranial bleeding			
Patients, *N* (%)	274 (2.4)	41 (1.8)	233 (2.5)
IPTW and index year adjusted, HR (95% CI)		**0.69 (0.48**–**0.98)**	Ref.
GI bleeding			
Patients, *N* (%)	1160 (10.0)	198 (8.6)	962 (10.4)
IPTW and index year adjusted, HR (95% CI)		**0.82 (0.69**–**0.96)**	Ref.
Recurrent VTE			
Patients, *N* (%)	736 (6.4)	152 (6.6)	584 (6.3)
IPTW and index year adjusted, HR (95% CI)		0.83 (0.69–1.002)	Ref.
All‐cause mortality			
Patients, *N* (%)	1187 (10.3)	231 (10.0)	956 (10.3)
IPTW and index year adjusted, HR (95% CI)		1.06 (0.91–1.24)	Ref.

*Note*: HR were generated using the intent‐to‐treat principle, defined by the medication first prescribed. We utilized a propensity score by modeling the probability of treatment with apixaban rather than warfarin for each cohort member using a logistic regression model including all available clinical covariates. We then weighted the individuals in the cohort using IPTW after truncating the weights at the 1st and 99th percentile. Bold values indicates statistically significant at *p* < 0.05.

Abbreviations: CI, confidence interval; GI, gastrointestinal; HR, hazard ratio; IPTW, inverse probability of treatment weighting; VTE, venous thromboembolism.

^a^ Total major bleeding is slightly less than fatal major bleeding and nonfatal major bleeding combined because some patients had a nonfatal major bleeding hospitalization followed by a fatal major bleeding hospitalization.

**Figure 1:**
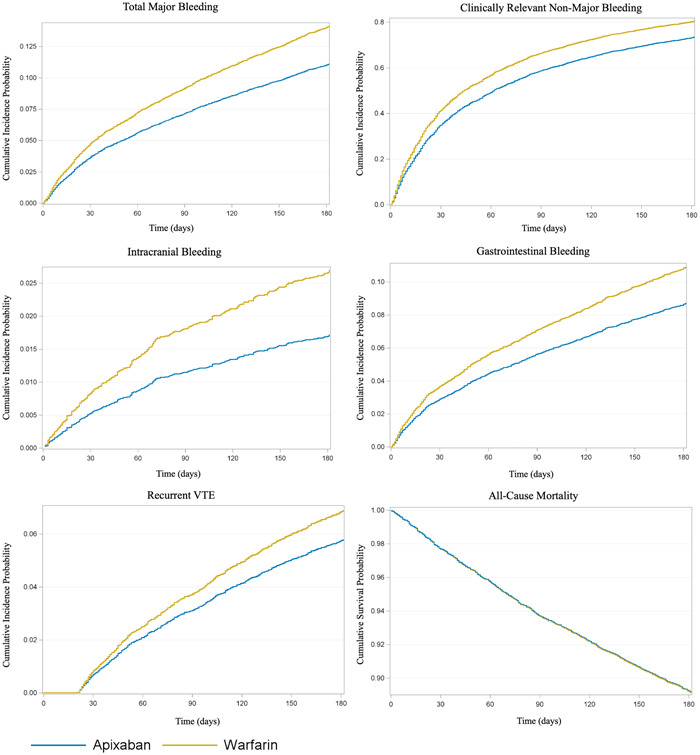
Cumulative incidence and survival probabilities for outcomes. Cumulative incidence and survival probabilities of prespecified outcomes to 6 months with direct adjustment for all covariates and index year.

The mean follow‐up time in the 6‐month, intent‐to‐treat analyses ranged from 145 days (clinically relevant nonmajor bleeding) to 163 days (all‐cause mortality) (Supporting Information: Appendix Table 5). Rates of major bleeding, recurrent VTE, and all‐cause mortality were primary analyses, while intracranial bleeding, GI bleeding, and clinically relevant nonmajor bleeding were secondary analyses. Relative to warfarin, apixaban was associated with a lower risk of major bleeding (HR 0.81, 95% CI: 0.70–0.94), intracranial bleeding (HR 0.69, 95% CI 0.48–0.98), and GI bleeding (HR 0.82, 95% CI 0.69–0.96) (Table 3 and Figure 1). Recurrent VTE and all‐cause mortality were not significantly different between the cohorts. Similarly, the rates of transfusion events (an exploratory analysis) were not significantly different between the two cohorts (Supporting Information: Appendix Table 6). In the intent‐to‐treat analysis, including only the first 3 months since initiating therapy, the bleeding outcomes again favored apixaban over warfarin, and the risk of recurrent VTE was lower in the apixaban group (Supporting Information: Appendix Table 7). Within the first month of treatment, only the rate of clinically relevant nonmajor bleeding was significantly lower in the apixaban group (Supporting Information: Appendix Table 8).

When we censored patients after discontinuation of the first assigned medication (as‐treated analysis), we found that the bleeding outcomes still favored apixaban over warfarin (major bleeding HR 0.75, 95% CI 0.63–0.89; GI bleeding HR 0.81, 95% CI 0.65–0.999). However, the rate of intracranial bleeding was not significantly different between the groups. Again, the rates of recurrent VTE and all‐cause mortality were not significantly different, though there was a trend toward a lower rate of recurrent VTE in the apixaban group (HR 0.77, 95% CI 0.59–1.01) (Supporting Information: Appendix Table 9).

The dosing subgroup exploratory analysis found that treatment with apixaban was associated with a lower risk of major bleeding in both the 5 mg dosing subgroup (HR 0.76, 95% CI 0.62–0.93) and 2.5 mg dosing subgroup compared to warfarin (HR 0.68, 95% CI 0.54–0.85) (Supporting Information: Appendix Tables 10–13). Relative to warfarin, treatment with 5 mg tablets of apixaban (10 mg total daily dose, the approved regimen) was also associated with a lower risk of clinically relevant nonmajor bleeding (HR 0.77, 95% CI 0.65–0.90) and recurrent VTE (HR 0.76, 95% CI 0.58–0.98). Finally, there was evidence of a lower risk of GI bleeding in the apixaban 2.5 mg subgroup relative to the 5 mg subgroup (HR 0.69, 95% CI 0.50–0.94). Using direct adjustment for covariates rather than IPTW did not significantly change our findings (Supporting Information: Appendix Table 14).

## DISCUSSION

In this retrospective cohort study, we found that the use of apixaban to treat acute VTE in ESKD increased dramatically between 2014 and 2018. In 2018, nearly an equal proportion of patients with ESKD received an apixaban‐based and warfarin‐based strategy for acute VTE. In our primary analysis, we found that apixaban was associated with lower bleeding compared to warfarin. Additionally, a signal suggesting a lower rate of recurrent VTE for apixaban relative to warfarin (a co‐primary analysis) was present. Our findings were generally robust in sensitivity analyses including those that censored patients when they were no longer exposed to the therapies of interest. Finally, in an exploratory analysis, we found that a relatively large percentage of the apixaban group was given an off‐label, lower dose in keeping with prior studies.^23^

The US Food and Drug Administration approved apixaban to treat AF in December 2012 and VTE in August 2014. Although the drug is partially renally cleared, there are no recommended dosing adjustments for patients with VTE and renal insufficiency.^12^ In patients with AF and ESKD, a retrospective cohort study showed a lower risk of major bleeding, thromboembolism, and mortality among apixaban‐treated patients compared to those treated with warfarin.^10^ A meta‐analysis showed a lower risk of mortality among apixaban‐treated patients with AF and ESKD compared to warfarin‐treated patients.^11^

Several studies also suggest the superiority of apixaban for the treatment of VTE relative to other agents.^24^, ^25^, ^26^, ^27^ An analysis of data from four commercial claims databases plus Medicare claims found that among patients with CKD, the risks of recurrent VTE, major bleeding, and clinically relevant nonmajor bleeding were lower in apixaban‐treated patients than in warfarin‐treated patients.^28^ Our analysis includes an additional 2 years of Medicare claims data (2017 and 2018) and focuses exclusively on patients with ESKD receiving dialysis. A recently published analysis using USRDS data to compare apixaban and warfarin to treat acute VTE in patients with ESKD also found a lower adjusted hazard ratio of major bleeding but also noted a significantly lower adjusted hazard ratio of recurrent VTE than in our study.^29^ Our analysis differed from that by Wetmore et al. in terms of covariates, the relationship of VTE diagnosis and index prescription timing for inclusion to cohort, the technique for ensuring patients were on maintenance dialysis, controlling for year of index prescription, and definition of recurrent VTE.

Our apixaban cohort was 26% smaller than that of Wetmore et al. which may be due to the fact that we rigorously excluded AF patients (they excluded AF patients only in a sensitivity analysis) and patients who were no longer on dialysis. We also used a more restrictive definition of recurrent VTE including only those patients with an event 21 days or more after the index event and requiring that the VTE code is in the primary position. Of note, our findings on recurrent VTE were not consistent with Wetmore et al. We included a broader range of bleeding outcomes, and we conducted a subanalysis for off‐label dosing of apixaban which was quite common: over 1/3 of apixaban‐treated patients received off‐label dosing. Finally, we accounted for temporal trends by adjusting for the year of enrollment in our modeling. Given that over the course of the study, apixaban use increased from less than 2% of ESKD patients treated for VTE to almost half, the risk for confounding due to secular trends is substantial.

The package inserts for both dabigatran and rivaroxaban indicate they are renally cleared and must be dose‐reduced in patients with CKD; the inserts make no dosing recommendations for VTE in ESKD.^30^, ^31^ Early pharmacokinetic evaluation of apixaban, however, allowed for it to be labeled and administered to patients with ESKD without dose adjustment.^32^ Recent single‐center studies evaluating the safety and effectiveness of apixaban for the treatment of VTE in severe CKD and ESKD suggest that it is associated with a lower bleeding risk than warfarin, with similar effectiveness.^7^, ^33^

Our findings suggest that physicians are increasingly comfortable using apixaban to treat acute VTE in patients with ESKD. Apixaban offers several potential advantages over warfarin including no requirement for bridging therapy with a heparin infusion and no need for therapeutic monitoring. We suspect that many physicians still use a warfarin‐based strategy in the ESKD population due to concerns about safety and effectiveness, affordability, concerns about reversibility, or based on defaulting to a “tried and true” strategy given limited early data with apixaban in the ESKD population.

Future studies might compare the healthcare utilization of an apixaban and warfarin‐based strategy for the ESKD population and evaluate the safety and effectiveness of an off‐label, 2.5 mg strategy for part of the VTE treatment course and for patient populations at higher risk for bleeding while on anticoagulants (e.g., those that meet criteria for dose reduction based on AF criteria or patients with a history of anticoagulant‐associated bleeding). Although we did not find any evidence that reduced dosing appears to be useful, dose reduction is not uncommon, and it is possible that in selected patients it leads to better outcomes.

There are several limitations of our study. The USRDS provides extensive information on Medicare fee‐for‐service beneficiaries with ESKD, and because the vast majority of patients with ESKD, including those under the age of 65, have Medicare as their primary payer, this data set includes nearly all Medicare fee‐for‐service beneficiaries with ESKD. However, it does not include claims from individuals in a Medicare Advantage plan. Thus, this population was not included in our analysis. We have no reason to suspect that these individuals would have different responses to anticoagulants than the included patients, although there may be demographic differences.

Additionally, USRDS (and the Medicare claims data that are included) does not provide information on what medications patients received during hospitalizations. Thus, we cannot characterize or control for initial anticoagulation, such as parenteral heparin products, during an index hospitalization. We suspect that the majority of patients in the warfarin cohort received an unfractionated heparin infusion as this is a standard of care in patients with ESKD.^34^ It is possible that many apixaban‐treated patients also received unfractionated heparin. Relatedly, we cannot say with certainty whether patients in the apixaban cohort received the recommended loading dose of 10 mg twice per day for the first 7 days of treatment due to the nature of medication claims data and the fact that there is not a 10 mg tablet (but instead patients take two 5 mg tablets, for a total dose of 10 mg twice per day during the loading period). Also, given our definition of recurrent VTE, any subsequent VTE events that occurred during a prolonged index hospitalization would not be included in the analysis. More generally, claims‐based definitions for VTE have been noted to have relatively low positive predictive values.^35^

Finally, there is a possibility of unmeasured confounding which is a risk with any study that is not a randomized clinical trial; we could not, for instance, account for over‐the‐counter aspirin use, although we have no reason to suspect this differed between apixaban and warfarin‐treated patients. Similarly, we could not adjust for body mass index differences, aside from including codes for obesity among our covariates. The fact that the two treatment groups were well‐matched prior to propensity score adjustment suggests that major residual confounding is unlikely.

These limitations not with standing, we conclude that apixaban was associated with a lower risk of bleeding and similar rates of recurrent VTE and all‐cause mortality compared to warfarin when used to treat acute VTE in patients with ESKD.

## CONFLICT OF INTEREST

Dr. Ardeshirrouhanifard's spouse is an employee of Eli Lilly and has equity in that company. Dr. Streiff has received honoraria from Pfizer and served on an advisory panel for them. He has also received research support from Janssen Pharmaceuticals and served on an advisory board for them through December 2020. Dr. Brotman has received payment for consulting for Bristol‐Myers Squibb. Dr. Deitelzweig has received payments for research, consulting, and speaking from Bristol‐Myers Squibb, Pfizer, and Alexion. He has also been an unpaid board member for the American College of Cardiology and the Society of Hospital Medicine. The remaining authors declare no conflict of interest.

## Supporting information

Supplementary information.
