# Supplementary material for: Safety and effectiveness of apixaban versus warfarin for acute venous thromboembolism in patients with end‐stage kidney disease: A national cohort study
Source: J Hosp Med. 2022 Aug 5;17(10):809–18. doi: 10.1002/jhm.12926 (PMC9804323; doi:10.1002/jhm.12926)
Supplement: Supplementary file 1 — Supplementary information. [file JHM-17-809-s001.docx]

**Online Only Supplement**

*Appendix Tables*

Table 1 – Venous thromboembolism inclusion codes – pages 2-5

Table 2 – Clinical covariates included in propensity score - pages 7-41

Table 3 – Definitions of outcomes - pages 42-45

Table 4 – Cohort creation – page 46

Table 5 – Mean follow-up days for outcomes – page 48

Table 6 – Counts of blood transfusion episodes per patient – page 49

Table 7 – Bleeding and thrombotic outcomes at 3 months, intention-to-treat – page 50

Table 8 – Bleeding and thrombotic outcomes at 1 month, intention-to-treat – page 51

Table 9 – Bleeding and thrombotic outcomes at 6 months, as-treated – page 52

Table 10 – Comparison of covariates between Apixaban dosing subgroups – page 53

Table 11 – Comparison of outcomes between Apixaban dosing subgroups – page 56

Table 12 – Comparison of outcomes between Apixaban 2.5mg Group and Warfarin – page 57

Table 13 – Comparison of outcomes between Apixaban 5mg Group and Warfarin – page 58

Table 14 – Comparison of outcomes in main analysis between IPTW and direct covariate

adjustment – page 59

*Appendix Figures*

Figure 1 - Study inclusion and exclusion criteria - page 6

Figure 2 - Change in Apixaban and Warfarin use by year – page 47

**Table 1. Venous Thromboembolism Inclusion Codes**

Note: ICD codes can be in either the inpatient or outpatient setting and in any position for cohort inclusion.

| **Diagnosis** | **ICD9 code** | **ICD10 code** |
| --- | --- | --- |
| Iatrogenic PE and infarction | 415.11 |  |
| Saddle embolus of pulmonary artery | 415.13 |  |
| Other pulmonary embolism and infarction | 415.19 |  |
| Acute venous embolism and thrombosis of unspecified deep vessels of lower extremity | 453.40 |  |
| Acute venous embolism and thrombosis of deep vessels of the proximal lower extremity | 453.41 |  |
| Acute venous embolism and thrombosis of deep vessels of distal lower extremity | 453.42 |  |
| Acute venous embolism and thrombosis of deep veins of upper extremity | 453.82 |  |
| Acute venous embolism and thrombosis of upper extremity, unspecified | 453.83 |  |
| Acute venous embolism and thrombosis of axillary veins | 453.84 |  |
| Acute venous embolism and thrombosis of subclavian veins | 453.85 |  |
| Acute venous embolism and thrombosis of internal jugular veins | 453.86 |  |
| Acute venous embolism and thrombosis of other thoracic veins | 453.87 |  |
| Acute venous embolism and thrombosis of other specified veins | 453.89 |  |
| Other venous embolism and thrombosis of inferior vena cava | 453.2 |  |
| Other venous embolism and thrombosis of renal vein | 453.3 |  |
| Other venous embolism and thrombosis of unspecified site | 453.9 |  |
| Phlebitis and thrombophlebitis of femoral vein | 451.11 |  |
| Phlebitis and thrombophlebitis of deep veins of lower extremities, other | 451.19 |  |
| Phlebitis and thrombophlebitis of lower extremities, unspecified | 451.2 |  |
| Phlebitis and thrombophlebitis of iliac vein | 451.81 |  |
| Phlebitis and thrombophlebitis of deep veins of upper extremities | 451.83 |  |
| Phlebitis and thrombophlebitis of upper extremities, unspecified | 451.84 |  |
| Phlebitis and thrombophlebitis of other sites | 451.89 |  |
| Phlebitis and thrombophlebitis of unspecified site | 451.9 |  |
| Deep phlebothrombosis antepartum | 671.3 |  |
| Deep phlebothrombosis postpartum | 671.4 |  |
| Other phlebitis and thrombosis in pregnancy and the puerperium | 671.5 |  |
| Obstetrical blood-clot embolism | 673.2 |  |
| Other obstetrical pulmonary embolism | 673.8 |  |
| Saddle embolus of pulmonary artery with acute cor pulmonale |  | I26.02 |
| Other pulmonary embolism with acute cor pulmonale |  | I26.09 |
| Saddle embolism of pulmonary artery without acute cor pulmonale |  | I26.92 |
| Single subsegmental pulmonary embolism without acute cor pulmonale |  | I26.93 |
| Multiple subsegmental pulmonary emboli without acute cor pulmonale |  | I26.94 |
| Other pulmonary embolism without acute cor pulmonale |  | I26.99 |
| Acute venous embolism and thrombosis of unspecified deep veins of the right lower extremity |  | I82.401 |
| Acute venous embolism and thrombosis of unspecified deep veins of the left lower extremity |  | I82.402 |
| Acute venous embolism and thrombosis of unspecified deep veins of bilateral lower extremities |  | I82.403 |
| Acute embolism and thrombosis of unspecified deep veins of unspecified lower extremity |  | I82.409 |
| Acute embolism and thrombosis of right femoral vein |  | I82.411 |
| Acute embolism and thrombosis of left femoral vein |  | I82.412 |
| Acute embolism and thrombosis of bilateral femoral veins |  | I82.413 |
| Acute embolism and thrombosis of unspecified femoral veins |  | I82.419 |
| Acute embolism and thrombosis of left iliac vein |  | I82.422 |
| Acute embolism and thrombosis of bilateral iliac veins |  | I82.423 |
| Acute embolism and thrombosis of unspecified iliac veins |  | I82.429 |
| Acute embolism and thrombosis of right popliteal vein |  | I82.431 |
| Acute embolism and thrombosis of left popliteal vein |  | I82.432 |
| Acute embolism and thrombosis of bilateral popliteal veins |  | I82.433 |
| Acute embolism and thrombosis of unspecified popliteal veins |  | I82.439 |
| Acute embolism and thrombosis of right tibial vein |  | I82.441 |
| Acute embolism and thrombosis of left tibial vein |  | I82.442 |
| Acute embolism and thrombosis of bilateral tibial veins |  | I82.443 |
| Acute embolism and thrombosis of unspecified tibial vein |  | I82.449 |
| Acute embolism and thrombosis of right peroneal vein |  | I82.451 |
| Acute embolism and thrombosis of left peroneal vein |  | I82.452 |
| Acute embolism and thrombosis of bilateral peroneal veins |  | I82.453 |
| Acute embolism and thrombosis of unspecified peroneal vein |  | I82.459 |
| Acute embolism and thrombosis of right calf muscular vein |  | I82.461 |
| Acute embolism and thrombosis of left calf muscular vein |  | I82.462 |
| Acute embolism and thrombosis of bilateral calf muscular vein |  | I82.463 |
| Acute embolism and thrombosis of unspecified calf muscular vein |  | I82.469 |
| Acute embolism and thrombosis of other specified deep vein of right lower extremity |  | I82.491 |
| Acute embolism and thrombosis of other specified deep vein of left lower extremity |  | I82.492 |
| Acute embolism and thrombosis of other specified deep vein of bilateral lower extremity |  | I82.493 |
| Acute embolism and thrombosis of other specified deep vein of unspecified lower extremity |  | I82.499 |
| Acute embolism and thrombosis of unspecified deep veins of right proximal lower extremity |  | I82.4Y1 |
| Acute embolism and thrombosis of unspecified deep veins of left proximal lower extremity |  | I82.4Y2 |
| Acute embolism and thrombosis of unspecified deep veins of bilateral proximal lower extremity |  | I82.4Y3 |
| Acute embolism and thrombosis of unspecified deep veins of unspecified proximal lower extremity |  | I82.4Y9 |
| Acute embolism and thrombosis of unspecified deep veins of right distal lower extremity |  | I82.4Z1 |
| Acute embolism and thrombosis of unspecified deep veins of left distal lower extremity |  | I82.4Z2 |
| Acute embolism and thrombosis of unspecified deep veins of bilateral distal lower extremity |  | I82.4Z3 |
| Acute embolism and thrombosis of unspecified deep veins of unspecified distal lower extremity |  | I82.4Z9 |
| Acute embolism and thrombosis of unspecified  veins of right upper extremity |  | I82.601 |
| Acute embolism and thrombosis of unspecified veins of left upper extremity |  | I82.602 |
| Acute embolism and thrombosis of unspecified veins of bilateral upper extremity |  | I82.603 |
| Acute embolism and thrombosis of unspecified veins of unspecified upper extremity |  | I82.609 |
| Acute embolism  and thrombosis of deep veins of right upper extremity |  | I82.621 |
| Acute embolism and thrombosis of deep veins of left upper extremity |  | I82.622 |
| Acute embolism and thrombosis of deep veins of bilateral upper extremity |  | I82.623 |
| Acute embolism and thrombosis of deep veins of unspecified upper extremity |  | I82.629 |
| Acute embolism and thrombosis of right axillary vein |  | I82.A11 |
| Acute embolism and thrombosis of left axillary vein |  | I82.A12 |
| Acute embolism and thrombosis of bilateral axillary vein |  | I82.A13 |
| Acute embolism and thrombosis of unspecified axillary vein |  | I82.A19 |
| Acute embolism and thrombosis of right subclavian vein |  | I82.B11 |
| Acute embolism and thrombosis of left subclavian vein |  | I82.B12 |
| Acute embolism and thrombosis of bilateral subclavian veins |  | I82.B13 |
| Acute embolism and thrombosis of unspecified subclavian vein |  | I82.B19 |
| Acute embolism and thrombosis of right internal jugular vein |  | I82.C11 |
| Acute embolism and thrombosis of left internal jugular vein |  | I82.C12 |
| Acute embolism and thrombosis of bilateral internal jugular vein |  | I82.C13 |
| Acute embolism and thrombosis of unspecified internal jugular vein |  | I82.C19 |
| Acute embolism and thrombosis of other specified veins |  | I82.890 |
| Acute embolism and thrombosis of unspecified vein |  | I82.90 |
| Embolism due to vascular prosthetic devices, implants, and grafts |  | T82.818A |
| Thrombosis due to vascular prosthetic devices, implants, and grafts |  | T82.868A |
| Acute embolism and thrombosis of superior vena cava |  | I82.210 |
| Acute embolism and thrombosis of inferior vena cava |  | I82.220 |
| Acute embolism and thrombosis of other thoracic veins |  | I82.290 |
| Embolism and thrombosis of renal vein |  | I82.3 |
| Phlebitis and thrombophlebitis of unspecified femoral vein |  | I80.10 |
| Phlebitis and thrombophlebitis of right femoral vein |  | I80.11 |
| Phlebitis and thrombophlebitis of left femoral vein |  | I80.12 |
| Phlebitis and thrombophlebitis of bilateral femoral vein |  | I80.13 |
| Phlebitis and thrombophlebitis of unspecified deep vessels of right lower extremity |  | I80.201 |
| Phlebitis and thrombophlebitis of unspecified deep vessels of left lower extremity |  | I80.202 |
| Phlebitis and thrombophlebitis of unspecified deep vessels of bilateral lower extremity |  | I80.203 |
| Phlebitis and thrombophlebitis of unspecified deep vessels of unspecified lower extremity |  | I80.209 |
| Phlebitis and thrombophlebitis of right iliac vein |  | I80.211 |
| Phlebitis and thrombophlebitis of left iliac vein |  | I80.212 |
| Phlebitis and thrombophlebitis of bilateral iliac vein |  | I80.213 |
| Phlebitis and thrombophlebitis of unspecified iliac vein |  | I80.219 |
| Phlebitis and thrombophlebitis of right popliteal vein |  | I80.221 |
| Phlebitis and thrombophlebitis of left popliteal vein |  | I80.222 |
| Phlebitis and thrombophlebitis of bilateral popliteal vein |  | I80.223 |
| Phlebitis and thrombophlebitis of unspecified popliteal vein |  | I80.229 |
| Phlebitis and thrombophlebitis of right tibial vein |  | I80.231 |
| Phlebitis and thrombophlebitis of left tibial vein |  | I80.232 |
| Phlebitis and thrombophlebitis of bilateral tibial vein |  | I80.233 |
| Phlebitis and thrombophlebitis of unspecified tibial vein |  | I80.239 |
| Phlebitis and thrombophlebitis of right peroneal vein |  | I80.241 |
| Phlebitis and thrombophlebitis of left peroneal vein |  | I80.242 |
| Phlebitis and thrombophlebitis of bilateral peroneal vein |  | I80.243 |
| Phlebitis and thrombophlebitis of unspecified peroneal vein |  | I80.249 |
| Phlebitis and thrombophlebitis of right calf muscular vein |  | I80.251 |
| Phlebitis and thrombophlebitis of left calf muscular vein |  | I80.252 |
| Phlebitis and thrombophlebitis of bilateral calf muscular vein |  | I80.253 |
| Phlebitis and thrombophlebitis of unspecified calf muscular vein |  | I80.259 |
| Phlebitis and thrombophlebitis of other deep vessels of right lower extremity |  | I80.291 |
| Phlebitis and thrombophlebitis of other deep vessels of left lower extremity |  | I80.292 |
| Phlebitis and thrombophlebitis of other deep vessels of lower extremity |  | I80.293 |
| Phlebitis and thrombophlebitis of other deep vessels of unspecified lower extremity |  | I80.299 |
| Deep phlebothrombosis in pregnancy unspecified trimester |  | O22.30 |
| Deep phlebothrombosis in pregnancy first trimester |  | O22.31 |
| Deep phlebothrombosis in pregnancy second trimester |  | O22.32 |
| Deep phlebothrombosis in pregnancy third trimester |  | O22.33 |
| Thromboembolism in pregnancy first trimester |  | O88.211 |
| Thromboembolism in pregnancy second trimester |  | O88.212 |
| Thromboembolism in pregnancy third trimester |  | O88.213 |
| Thromboembolism in pregnancy unspecified trimester |  | O88.219 |
| Thromboembolism in childbirth |  | O88.22 |
| Thromboembolism in the puerperium |  | O88.23 |
| Other embolism in pregnancy first trimester |  | O88.811 |
| Other embolism in pregnancy second trimester |  | O88.812 |
| Other embolism in pregnancy third trimester |  | O88.813 |
| Other embolism in pregnancy unspecified trimester |  | O88.819 |
| Other embolism in childbirth |  | O88.82 |
| Other embolism in the puerperium |  | O88.83 |

**Figure 1. Study Inclusion and Exclusion Criteria**

ESKD – End Stage Kidney Disease

LMWH – Low Molecular Weight Heparin

VTE – Venous Thromboembolism

**Table 2. Clinical Covariates Included in Propensity Score**

| Covariate | ICD9 diagnosis and procedure codes / HCPC / CPT / DRG codes^1^ | ICD10 diagnosis and procedure codes / HCPC / CPT / DRG codes^1^ | Timeframe | Care Setting | Source |
| --- | --- | --- | --- | --- | --- |
| Anemia | 280.0, 280.1, 280.8, 280.9, 281.0, 281.1, 281.2, 281.3, 281.4, 281.8, 281.9, 282.0, 282.1, 282.2, 282.3, 282.40, 282.41, 282.42, 282.43, 282.44, 282.45, 282.46, 282.47, 282.49, 282.5, 282.60, 282.61, 282.62, 282.63, 282.64, 282.68, 282.69, 282.7, 282.8, 282.9, 283.0, 283.10, 283.11, 283.19, 283.2, 283.9, 284.01, 284.09, 284.11, 284.12, 284.19, 284.2, 284.81, 284.89, 284.9, 285.0, 285.1, 285.21, 285.22, 285.29, 285.3, 285.8, 285.9 | D50.0, D50.1, D50.8, D50.9, D51.0, D51.1, D51.2, D51.3, D51.8, D51.9, D52.0, D52.1, D52.8, D52.9, D53.0, D53.1, D53.2, D53.8, D53.9, D55.0, D55.1, D55.2, D55.3, D55.8, D55.9, D56.0, D56.1, D56.2, D56.3, D56.4, D56.5, D56.8, D56.9, D57.00, D57.01, D57.02, D57.03, D57.09, D57.1, D57.20, D57.211, D57.212, D57.213, D57.218, D57.219, D57.3, D57.40, D57.411, D57.412, D57.413, D57.418, D57.419, D57.42, D57.431, D57.432, D57.433, D57.438, D57.439, D57.44, D57.451, D57.452, D57.453, D57.458, D57.459, D57.80, D57.811, D57.812, D57.813, D57.818, D57.819, D58.0, D58.1, D58.2, D58.8, D58.9, D59.0, D59.1, D59.10, D59.11, D59.12, D59.13, D59.19, D59.2, D59.3, D59.4, D59.5, D59.6, D59.8, D59.9, D60.0, D60.1, D60.8, D60.9, D61.01, D61.09, D61.1, D61.2, D61.3, D61.810, D61.811, D61.818, D61.82, D61.89, D61.9, D62, D63.0, D63.1, D63.8, D64.0, D64.1, D64.2, D64.3, D64.4, D64.81, D64.89, D64.9 | Up to 6 months before index date | At least 1 IP, SNF, HHA, HOP, or Carrier claim | CCW |
| Diabetes | 249.00, 249.01, 249.10, 249.11, 249.20, 249.21, 249.30, 249.31, 249.40, 249.41, 249.50, 249.51, 249.60, 249.61, 249.70, 249.71, 249.80, 249.81, 249.90, 249.91, 250.00, 250.01, 250.02, 250.03, 250.10, 250.11, 250.12, 250.13, 250.20, 250.21, 250.22, 250.23, 250.30, 250.31, 250.32, 250.33, 250.40, 250.41, 250.42, 250.43, 250.50, 250.51, 250.52, 250.53, 250.60, 250.61, 250.62, 250.63, 250.70, 250.71, 250.72, 250.73, 250.80, 250.81, 250.82, 250.83, 250.90, 250.91, 250.92, 250.93,  357.2, 362.01, 362.02, 362.03,  362.04, 362.05, 362.06, 366.41 | E08.00, E08.01, E08.10, E08.11, E08.21, E08.22, E08.29, E08.311, E08.319, E08.321, E08.3211, E08.3212, E08.3213, E08.3219, E08.329, E08.3291, E08.3292, E08.3293, E08.3299, E08.331, E08.3311, E08.3312, E08.3313, E08.3319, E08.339, E08.3391, E08.3392, E08.3393, E08.3399, E08.341, E08.3411, E08.3412, E08.3413, E08.3419, E08.349, E08.3491, E08.3492, E08.3493, E08.3499, E08.351, E08.3511, E08.3512, E08.3513, E08.3519, E08.3521, E08.3522, E08.3523, E08.3529, E08.3531, E08.3532, E08.3533, E08.3539, E08.3541, E08.3542, E08.3543, E08.3549, E08.3551, E08.3552, E08.3553, E08.3559, E08.359, E08.3591, E08.3592, E08.3593, E08.3599, E08.36, E08.37X1, E08.37X2, E08.37X3, E08.37X9, E08.39, E08.40, E08.41, E08.42, E08.43, E08.44, E08.49, E08.51, E08.52, E08.59, E08.610, E08.618, E08.620, E08.621, E08.622, E08.628, E08.630, E08.638, E08.641, E08.649, E08.65, E08.69, E08.8, E08.9, E09.00, E09.01, E09.10, E09.11, E09.21, E09.22, E09.29, E09.311, E09.319, E09.321, E09.3211, E09.3212, E09.3213, E09.3219, E09.329, E09.3291, E09.3292, E09.3293, E09.3299, E09.331, E09.3311, E09.3312, E09.3313, E09.3319, E09.339, E09.3391, E09.3392, E09.3393, E09.3399, E09.341, E09.3411, E09.3412, E09.3413, E09.3419, E09.349, E09.3491, E09.3492, E09.3493, E09.3499, E09.351, E09.3511, E09.3512, E09.3513, E09.3519, E09.3521, E09.3522, E09.3523, E09.3529, E09.3531, E09.3532, E09.3533, E09.3539, E09.3541, E09.3542, E09.3543, E09.3549, E09.3551, E09.3552, E09.3553, E09.3559, E09.359, E09.3591, E09.3592, E09.3593, E09.3599, E09.36, E09.37X1, E09.37X2, E09.37X3, E09.37X9, E09.39, E09.40, E09.41, E09.42, E09.43, E09.44, E09.49, E09.51, E09.52, E09.59, E09.610, E09.618, E09.620, E09.621, E09.622, E09.628, E09.630, E09.638, E09.641, E09.649, E09.65, E09.69, E09.8, E09.9, E10.10, E10.11, E10.21, E10.22, E10.29, E10.311, E10.319, E10.321, E10.3211, E10.3212, E10.3213, E10.3219, E10.329, E10.3291, E10.3292, E10.3293, E10.3299, E10.331, E10.3311, E10.3312, E10.3313, E10.3319, E10.339, E10.3391, E10.3392, E10.3393, E10.3399, E10.341, E10.3411, E10.3412, E10.3413, E10.3419, E10.349, E10.3491, E10.3492, E10.3493, E10.3499, E10.351, E10.3511, E10.3512, E10.3513, E10.3519, E10.3521, E10.3522, E10.3523, E10.3529, E10.3531, E10.3532, E10.3533, E10.3539, E10.3541, E10.3542, E10.3543, E10.3549, E10.3551, E10.3552, E10.3553, E10.3559, E10.359, E10.3591, E10.3592, E10.3593, E10.3599, E10.36, E10.37X1, E10.37X2, E10.37X3, E10.37X9, E10.39, E10.40, E10.41, E10.42, E10.43, E10.44, E10.49, E10.51, E10.52, E10.59, E10.610, E10.618, E10.620, E10.621, E10.622, E10.628, E10.630, E10.638, E10.641, E10.649, E10.65, E10.69, E10.8, E10.9, E11.00, E11.01, E11.10, E11.11, E11.21, E11.22, E11.29, E11.311, E11.319, E11.321, E11.3211, E11.3212, E11.3213, E11.3219, E11.329, E11.3291, E11.3292, E11.3293, E11.3299, E11.331, E11.3311, E11.3312, E11.3313, E11.3319, E11.339, E11.3391, E11.3392, E11.3393, E11.3399, E11.341, E11.3411, E11.3412, E11.3413, E11.3419, E11.349, E11.3491, E11.3492, E11.3493, E11.3499, E11.351, E11.3511, E11.3512,  E11.3513, E11.3519, E11.3521, E11.3522, E11.3523, E11.3529, E11.3531, E11.3532, E11.3533, E11.3539, E11.3541, E11.3542, E11.3543, E11.3549, E11.3551, E11.3552, E11.3553, E11.3559, E11.359, E11.3591, E11.3592, E11.3593, E11.3599, E11.36, E11.37X1, E11.37X2, E11.37X3, E11.37X9, E11.39, E11.40, E11.41, E11.42, E11.43, E11.44, E11.49, E11.51, E11.52, E11.59, E11.610, E11.618, E11.620, E11.621, E11.622, E11.628, E11.630, E11.638, E11.641, E11.649, E11.65, E11.69, E11.8, E11.9, E13.00, E13.01, E13.10, E13.11, E13.21, E13.22, E13.29, E13.311, E13.319, E13.321, E13.3211, E13.3212, E13.3213, E13.3219, E13.329, E13.3291, E13.3292, E13.3293, E13.3299, E13.331, E13.3311, E13.3312, E13.3313, E13.3319, E13.339, E13.3391, E13.3392, E13.3393, E13.3399, E13.341, E13.3411, E13.3412, E13.3413, E13.3419, E13.349, E13.3491, E13.3492, E13.3493, E13.3499, E13.351, E13.3511, E13.3512, E13.3513, E13.3519, E13.3521, E13.3522, E13.3523, E13.3529, E13.3531, E13.3532, E13.3533, E13.3539, E13.3541, E13.3542, E13.3543, E13.3549, E13.3551, E13.3552, E13.3553, E13.3559, E13.359, E13.3591, E13.3592, E13.3593, E13.3599, E13.36, E13.39, E13.40, E13.41, E13.42, E13.43, E13.44, E13.49, E13.51, E13.52, E13.59, E13.610, E13.618, E13.620, E13.621, E13.622, E13.628, E13.630, E13.638, E13.641, E13.649, E13.65, E13.69, E13.8, E13.9 | Up to 6 months before index date | At least 1 IP, SNF, HHA or 2 HOP or Carrier claims | CCW |
| Hypertension | 362.11, 401.0, 401.1, 401.9, 402.00, 402.01, 402.10, 402.11, 402.90, 402.91, 403.00, 403.01, 403.10, 403.11, 403.90, 403.91, 404.00, 404.01, 404.02, 404.03, 404.10, 404.11, 404.12, 404.13, 404.90, 404.91, 404.92, 404.93, 405.01, 405.09, 405.11, 405.19, 405.91, 405.99, 437.2 | H35.031, H35.032, H35.033, H35.039, I10, I11.0, I11.9, I12.0, I12.9, I13.0, I13.10, I13.11, I13.2, I15.0, I15.1, I15.2, I15.8, I15.9, I67.4, N26.2 | Up to 6 months before index date | At least 1 IP, SNF, HHA or 2 HOP or Carrier claims | CCW |
| Ischemic Heart Disease | 410.00, 410.01, 410.02, 410.10, 410.11, 410.12, 410.20, 410.21, 410.22, 410.30, 410.31, 410.32, 410.40, 410.41, 410.42, 410.50, 410.51, 410.52, 410.60, 410.61, 410.62, 410.70, 410.71, 410.72, 410.80, 410.81, 410.82, 410.90, 410.91, 410.92, 411.0, 411.1, 411.81, 411.89, 412, 413.0, 413.1, 413.9, 414.00, 414.01, 414.02, 414.03, 414.04, 414.05, 414.06, 414.07, 414.12, 414.2, 414.3, 414.4, 414.8, 414.9 | I20.0, I20.1, I20.8, I20.9, I21.01, I21.02, I21.09, I21.11, I21.19, I21.21, I21.29, I21.3, I21.4, I21.A1, I21.A9, I22.0, I22.1, I22.2, I22.8, I22.9, I23.0, I23.1, I23.2, I23.3, I23.4, I23.5, I23.6, I23.7, I23.8, I24.0, I24.1, I24.8, I24.9, I25.10, I25.110, I25.111, I25.118, I25.119, I25.2, I25.3, I25.41, I25.42, I25.5, I25.6, I25.700, I25.701, I25.708, I25.709, I25.710, I25.711, I25.718, I25.719, I25.720, I25.721, I25.728, I25.729, I25.730, I25.731, I25.738, I25.739, I25.750, I25.751, I25.758, I25.759, I25.760, I25.761, I25.768, I25.769, I25.790, I25.791, I25.798, I25.799, I25.810, I25.811, I25.812, I25.82, I25.83, I25.84, I25.89, I25.9 | Up to 6 months before index date | At least 1 IP, SNF, HHA, HOP, or Carrier claim | CCW |
| Obesity | 278.0, 278.00, 278.01, 278.03, V85.3, V85.30, V85.31, V85.32, V85.33, V85.34, V85.35, V85.36, V85.37, V85.38, V85.39, V85.4, V85.41, V85.42, V85.43, V85.44, V85.45 | E66.01, E66.09, E66.1, E66.2, E66.8, E66.9, Z68.30, Z68.31, Z68.32, Z68.33, Z68.34, Z68.35, Z68.36, Z68.37, Z68.38, Z68.39, Z68.41, Z68.42, Z68.43, Z68.44, Z68.45 | Any time in claims data prior to index date | At least 1 IP or 2 non-IP claims (or if BMI ≥ 30 was coded in USRDS) | CCW definition and USRDS data includes BMI (we used cutoff of ≥ 30) |
| Peptic Ulcer Disease | 533.1X, 533.3X, 533.5X,  533.7X, 533.9X , 530.20, 531.1X, 531.3X, 531.5X, 531.7X, 531.9X, 532.1X, 532.3X, 532.5X, 532.7X,  532.9X, 534.1X, 534.3X, 534.5X, 534.7X, 534.9X, | K25.1, K25.3, K25.5, K25.7, K25.9, K26.1, K26.3, K26.5, K26.7, K26.9, K27.1, K27.3,  K27.5, K27.7, K27.9, K28.1, K28.3, K28.5, K28.7, K28.9 | Up to 6 months before index date | At least 1 IP, HOP, SNF, or carrier claim | Investigator review of ICD codes for peptic ulcer disease |
| Pulmonary Hypertension | 416.0, 416.8 | I27.0, I27.20, I27.21, I27.22, I27.23, I27.24, I27.29 | Up to 6 months before index date | At least 1 IP, HOP, SNF, or carrier claim | Investigator review of ICD codes |
| Tobacco use | ICD: 305.1, 649.00, 649.01, 649.02, 649.03, 649.04, 989.84  HCPC: 99406, 99407 | ICD: F17.200, F17.201, F17.203, F17.208, F17.209, F17.210, F17.211, F17.213, F17.218, F17.219, F17.220, F17.221, F17.223, F17.228, F17.229, F17.290, F17.291, F17.293, F17.298, F17.299, O99.330, O99.331, O99.332, O99.333, O99.334, O99.335, T65.211A, T65.212A, T65.213A, T65.214A, T65.221A, T65.222A, T65.223A, T65.224A, T65.291A, T65.292A, T65.293A, T65.294A, Z72.0  HCPC: 99406, 99407, G9276, G9458 | Any time in claims data prior to index date | At least 1 inpatient or 2 other non-drug claims of any service type with diagnosis codes or 1 HCPCS code claim of any type or coded as a USRDS variable | CCW definition and USRDS includes a tobacco use indicator variable |
| Prior VTE | 415.11, 415.13, 415.19,  453.4, 453.41, 453.42,  453.82, 453.83, 453.84,  453.85, 453.86, 453.87,  453.89, 453.2, 453.3,  453.9, 451.11, 451.19,  451.2, 451.81, 451.83,  451.84, 451.89, 451.9,  671.3, 671.4, 671.5,  673.2, 673.8, 452,  453.5, 453.51, 453.52,  453.71, 453.72, 453.73,  453.74, 453.75, 453.76,  453.77, 453.79, 416.2 | I26.02, I26.09, I26.92, I26.93, I26.94, I26.99, I82.401, I82.402, I82.403, I82.409, I82.411, I82.412, I82.413, I82.419, I82.421, I82.422, I82.423, I82.429, I82.431, I82.432,  I82.433, I82.439, I82.441, I82.442, I82.443, I82.449, I82.451, I82.452,  I82.453, I82.459, I82.461, I82.462,  I82.463, I82.469, I82.491, I82.492,  I82.493, I82.499, I82.4Y1, I82.4Y2, I82.4Y3, I82.4Y9,  I82.4Z1, I82.4Z2, I82.4Z3, I82.4Z9, I82.601, I82.602,  I82.603, I82.609, I82.621, I82.622,  I82.623, I82.629, I82.A11, I82.A12, I82.A13, I82.A19,  I82.B11, I82.B12, I82.B13, I82.B19, I82.C11, I82.C12,  I82.C13, I82.C19, I82.890, I82.90,  T82.818A, T82.868A, I82.210, I82.220, I82.290, I82.3, I80.10, I80.11, I80.12, I80.13, I80.201, I80.202, I80.203, I80.209,  I80.211, I80.212, I80.213, I80.219,  I80.221, I80.222, I80.223, I80.229,  I80.231, I80.232, I80.233, I80.239,  I80.241, I80.242, I80.243, I80.249,  I80.251, I80.252, I80.253, I80.259,  I80.291, I80.292, I80.293, I80.299,  O22.30, O22.31, O22.32, O22.33,  O88.211, O88.212, O88.213, O88.219, O88.22, O88.23, O88.811, O88.812, O88.813, O88.819, O88.82, O88.83,  I81, I82.211, I82.221, I82.291, I82.501, I82.502, I82.503, I82.509, I82.511, I82.512, I82.513, I82.519, I82.521, I82.522, I82.523, I82.529, I82.531, I82.532, I82.533, I82.539, I82.541, I82.542, I82.543, I82.549, I82.551, I82.552, I82.553, I82.559, I82.561, I82.562, I82.563, I82.569, I82.591, I82.592, I82.593, I82.599, I82.5Y1, I82.5Y2, I82.5Y3, I82.5Y9, I82.5Z1, I82.5Z2, I82.5Z3, I82.5Z9, I82.701, I82.702, I82.703, I82.709, I82.721, I82.722, I82.723, I82.729, I82.A21, I82.A22, I82.A23, I82.A29, I82.B21, I82.B22, I82.B23,  I82.B29, I82.C21, I82.C22, I82.C23, I82.C29, I82.891,  I82.91, I27.82, | From six months before to 1 month before index event | At least 1 IP, HOP, SNF, or carrier claim | Investigator review of ICD codes |
| Prior GI bleeding | 533.0X, 533.2X, 533.4X,  533.6X, 530.82, 530.21,  531.0X, 531.2X,  531.4X, 531.6X, 532.0X,  532.2X, 532.4X, 532.6X,  534.0X, 534.2X, 534.4X,  534.6X, 535.01, 535.11, 535.21, 535.31, 535.41, 535.51, 535.61, 535.71,  578.X, 562.02, 562.03,  562.12, 562.13, 569.3,  569.85, 537.83, 569.86 | K20.81, K20.91, K21.01, K22.11,  K25.0, K25.2, K25.4, K25.6, K26.0, K26.2, K26.4, K26.6, K27.0, K27.2, K27.4, K27.6,  K28.0, K28.2, K28.4, K28.6, K29.01, K29.21, K29.31, K29.41, K29.51, K29.61, K29.71, K29.81, K29.91, K31.811, K31.82, K52.21, K57.01, K57.11, K57.13, K57.21,  K57.31, K57.33, K57.41, K57.51,  K57.53, K57.81, K57.91, K57.93,  K62.5, K92.0, K92.1, K92.2 | Up to 6 months before index date | At least 1 IP, HOP, SNF, or carrier claim | Investigator review of ICD codes |
| Peripheral arterial disease | 440.2X, 440.3X, 440.4, 443.89, 443.9 | I70.2X, I70.25, I70.3X  I70.4X, I70.5X, I70.6X  I70.7X, I70.92, I73.89  I73.9 | Up to 6 months before the index date | At least 1 IP, HOP, SNF, or carrier claim | Investigator review of ICD codes |
| Liver disease (except viral hepatitis) | 570, 571, 571.0, 571.1, 571.2, 571.3, 571.5, 571.6, 571.8, 571.9, 572, 572.0, 572.1, 572.2, 572.3, 572.4, 572.8, 573, 573.0, 573.4, 573.5, 573.8, 573.9, 576.1, 789.1, V42.7  ICD9 Procedure codes:  42.91, 44.91, 54.91, 96.06 | K70.0, K70.10, K70.11, K70.2, K70.30, K70.31, K70.40, K70.41, K70.9, K71.0, K71.11, K71.7, K71.8, K71.9, K72.00, K72.01, K72.10, K72.11, K72.90, K72.91, K74.0, K74.00, K74.01, K74.02, K74.1, K74.2, K74.3, K74.4, K74.5, K74.60, K74.69, K75.0, K75.1, K75.81, K75.89, K75.9, K76.0, K76.1, K76.2, K76.3, K76.5, K76.6, K76.7, K76.81, K76.89, K76.9, K77, K80.30, K80.31, K80.32, K80.33, K80.34, K80.35, K80.36, K80.37, K83.0, R16.0, R16.2, Z48.23, Z94.4  ICD10 Procedure codes: 06L20ZZ, 06L23ZZ, 06L24ZZ, 06L30ZZ, 06L33ZZ, 06L34ZZ, 0DL57DZ, 0DL58DZ, 0D9S30Z, 0D9S3ZZ, 0D9S40Z, 0D9S4ZZ, 0D9T30Z, 0D9T3ZZ, 0D9T40Z, 0D9T4ZZ, 0D9V30Z, 0D9V3ZZ, 0D9V40Z, 0D9V4ZZ, 0D9W30Z, 0D9W3ZZ, 0D9W40Z, 0D9W4ZZ, 0W9F30Z, 0W9F3ZZ, 0W9F40Z, 0W9F4ZZ, 0W9G30Z, 0W9G3ZZ, 0W9G40Z, 0W9G4ZZ, 0W9J30Z, 0W9J3ZZ | Up to 6 months before the index date | At least 1 IP OR 2 non-IP claims with diagnosis codes.  Procedures must have at least one associated liver disease diagnosis | CCW |
| Active Malignancy | 140-165  170-172  174-176  179-209  238.4  238.7X | C00-C26  C30-41  C43  C4A  C45-80  C7A  C7B  C81-96  D45-D46 | Up to 6 months before the index date | At least 1 IP, HOP, SNF, or carrier claim | Reviewed all ICD codes for non-basal, non-squamous cell skin cancers and excluded carcinoma in situ, squamous cell skin cancer, basal cell skin cancer, benign neoplasms, neoplasms of uncertain behavior, and neoplasms of unspecified nature with the exception of the lymphatic and hemopoietic ones (Similar to NEJM Edoxaban Trial, Raskob et al) |
| Prescence of central catheter | 38.93, 38.97, V58.81  CPT codes: 36556, 36558, 36561, 36563, 36565, 36566, 36569,  36571, 36573, 36580, 36584, 36575, 36581, 36593, 36595, 36596 | Z45.2  CPT codes: 36556, 36558, 36561, 36563, 36565, 36566, 36569,  36571, 36573, 36580, 36584, 36575, 36581, 36593, 36595, 36596 | Up to 6 months before the index date | At least 1 IP, HOP, SNF, or carrier claim | Based on investigator review and Yusef et al article |
| Recent trauma | 800-848, 850-854,  860-887, 890-897,  900-904, 910-929,  950-957, 959 | S00-S99, T07, T14 | Up to 30 days before the index date | At least 1 IP, HOP, SNF, or carrier claim | Natasi et al |
| Recent surgery | DRG codes:  001-008, 010-014  014, 016-017, 020-042  113-117, 129-139,163-168, 215-229, 231-236, 239-274, 319-320,  326-358, 405-425,  453-483, 485-489,  492-520, 570-585,  614-618, 619-630,  652-675, 707-718,  734-750, 768-770,  783-788, 796-804,  817-830, 853-858, 876,  901-909, 927-929,  939-941, 955-959  969-970 | DRG codes:  001-008, 010-014  014, 016-017, 020-042  113-117, 129-139,163-168, 215-229, 231-236, 239-274, 319-320,  326-358, 405-425,  453-483, 485-489,  492-520, 570-585,  614-618, 619-630,  652-675, 707-718,  734-750, 768-770,  783-788, 796-804,  817-830, 853-858, 876,  901-909, 927-929,  939-941, 955-959  969-970 | Up to 60 days before the index date | At least 1 IP claim | Burwen et al |
| Heart failure | 398.91, 402.01, 402.11, 402.91, 404.01, 404.03, 404.11, 404.13, 404.91, 404.93, 428.0, 428.1, 428.20, 428.21, 428.22, 428.23, 428.30, 428.31, 428.32, 428.33, 428.40, 428.41, 428.42, 428.43, 428.9 | I09.81, I11.0, I13.0, I13.2, I50.1, I50.20, I50.21, I50.22, I50.23, I50.30, I50.31, I50.32, I50.33, I50.40, I50.41, I50.42, I50.43, I50.810, I50.811, I50.812, I50.813, I50.814, I50.82, I50.83, I50.84, I50.89, I50.9 | Up to 6 months before the index date | At least 1 inpatient, HOP, or Carrier claim | CCW |
| Prior major bleeding | See Outcomes Table | See Outcomes Table | Up to 6 months before the index date | At least 1 hospital inpatient or HOP claim | Same criteria as for defined in the outcomes table as defined by Cunningham et al (except for bleeding associated with death) |
| Stroke or TIA | 430, 431, 433.01, 433.11, 433.21, 433.31, 433.81, 433.91, 434.00, 434.01, 434.10, 434.11, 434.90, 434.91, 435.0, 435.1, 435.3, 435.8, 435.9, 436, 997.02  EXCLUSION: If any of the qualifying claims have: 800 <= DX Code <= 804.9, 850 <= DX Code <= 854.1 in any DX position OR DX V57xx as the principal DX Code, then EXCLUDE. | G45.0, G45.1, G45.2, G45.8, G45.9, G46.0, G46.1, G46.2, G46.3, G46.4, G46.5, G46.6, G46.7, G46.8, G97.31, G97.32, I60.00, I60.01, I60.02, I60.10, I60.11, I60.12, I60.20, I60.21, I60.22, I60.30, I60.31, I60.32, I60.4, I60.50, I60.51, I60.52, I60.6, I60.7, I60.8, I60.9, I61.0, I61.1, I61.2, I61.3, I61.4, I61.5, I61.6, I61.8, I61.9, I63.00, I63.011, I63.012, I63.013, I63.019, I63.02, I63.031, I63.032, I63.039, I63.09, I63.10, I63.111, I63.112^11–14^, I63.119, I63.12, I63.131, I63.132, I63.139, I63.19, I63.20, I63.211, I63.212, I63.213, I63.219, I63.22, I63.231, I63.232, I63.233, I63.239, I63.29, I63.30, I63.311, I63.312, I63.313, I63.319, I63.321, I63.322, I63.323, I63.329, I63.331, I63.332, I63.333, I63.339, I63.341, I63.342, I63.343, I63.349, I63.39, I63.40, I63.411, I63.412, I63.413, I63.419, I63.421, I63.422, I63.423, I63.429, I63.431, I63.432, I63.433, I63.439, I63.441, I63.442, I63.443, I63.449, I63.49, I63.50, I63.511, I63.512, I63.513, I63.519, I63.521, I63.522, I63.523, I63.529, I63.531, I63.532, I63.533, I63.539, I63.541, I63.542, I63.543, I63.549, I63.59, I63.6, I63.8, I63.9, I66.01, I66.02, I66.03, I66.09, I66.11, I66.12, I66.13, I66.19, I66.21, I66.22, I66.23, I66.29, I66.3, I66.8, I66.9, I67.841, I67.848, I67.89, I97.810, I97.811, I97.820, I97.821  EXCLUSION: If any of the qualifying claims have any of the following codes in any DX position then EXCLUDE: S01.90XA, S02.0XXA, S02.0XXB, S02.10XA, S02.10XB, S02.101A, S02.101B, S02.102A, S02.102B, S02.109A, S02.109B, S02.11GA, S02.11GB, S02.11HA, S02.11HB, S02.110A, S02.111A, S02.112A, S02.113A, S02.110B, S02.111B, S02.112B, S02.113B, S02.118A, S02.118B, S02.119A, S02.119B, S02.121A, S02.121B, S02.121D, S02.121G, S02.121K, S02.121S, S02.122A, S02.122B, S02.122D, S02.122G, S02.122K, S02.122S, S02.129A, S02.129B, S02.129D, S02.129G, S02.129K, S02.129S, S02.19XA, S02.19XB, S02.2XXA, S02.2XXB, S02.3XXA, S02.30XA, S02.3XXB, S02.30XB, S02.31XA, S02.31XB, S02.32XA, S02.32XB, S02.40AA, S02.40AB, S02.40BA, S02.40BB, S02.40CA, S02.40CB, S02.40DA, S02.40DB, S02.40EA, S02.40EB, S02.40FA, S02.40FB, S02.400A, S02.400B, S02.401A, S02.401B, S02.402A, S02.402B, S02.411A, S02.411B, S02.412A, S02.412B, S02.413A, S02.413B, S02.42XA, S02.42XB, S02.600A, S02.600B, S02.601A, S02.601B, S02.602A, S02.602B, S02.609A, S02.609B, S02.61XA, S02.610A, S02.61XB, S02.610B, S02.611A, S02.611B, S02.612A, S02.612B, S02.62XA, S02.620A, S02.62XB, S02.620B, S02.621A, S02.621B, S02.622A, S02.622B, S02.63XA, S02.630A, S02.63XB, S02.630B, S02.631A, S02.631B, S02.632A, S02.632B, S02.64XA, S02.640A, S02.64XB, S02.640B, S02.641A, S02.641B, S02.642A, S02.642B, S02.65XA, S02.650A, S02.65XB, S02.650B, S02.651A, S02.651B, S02.652A, S02.652B, S02.66XA, S02.66XB, S02.67XA, S02.670A, S02.67XB, S02.670B, S02.671A, S02.671B, S02.672A, S02.672B, S02.69XA, S02.69XB, S02.8XXA, S02.80XA, S02.8XXB, S02.80XB, S02.81XA, S02.81XB, S02.82XA, S02.82XB, S02.831A, S02.831B, S02.831D, S02.831G, S02.831K,  S02.831S, S02.832A, S02.832B, S02.832D, S02.832G, S02.832K, S02.832S, S02.839A, S02.839B, S02.839D, S02.839G, S02.839K, S02.839S, S02.841A, S02.841B, S02.841D, S02.841G, S02.841K, S02.841S, S02.842A, S02.842B, S02.842D, S02.842G, S02.842K, S02.842S, S02.849A, S02.849B, S02.849D, S02.849G, S02.849K, S02.849S, S02.85XA, S02.85XB, S02.85XD, S02.85XG, S02.85XK, S02.85XS, S02.91XA, S02.91XB, S02.92XA, S02.92XB, S06.0X0A, S06.0X1A, S06.0X2A, S06.0X3A, S06.0X4A, S06.0X5A, S06.0X6A, S06.0X7A, S06.0X8A, S06.0X9A, S06.1X0A, S06.1X1A, S06.1X2A, S06.1X3A, S06.1X4A, S06.1X5A, S06.1X6A, S06.1X7A, S06.1X8A, S06.1X9A, S06.2X0A, S06.2X1A, S06.2X2A, S06.2X3A, S06.2X4A, S06.2X5A, S06.2X6A, S06.2X7A, S06.2X8A, S06.2X9A, S06.2X0B, S06.2X1B, S06.2X2B, S06.2X3B, S06.2X4B, S06.2X5B, S06.2X6B, S06.2X7B, S06.2X8B, S06.2X9B, S06.300A, S06.301A, S06.302A, S06.303A, S06.304A, S06.305A, S06.306A, S06.307A, S06.308A, S06.309A, S06.310A, S06.311A, S06.312A, S06.313A, S06.314A, S06.315A, S06.316A, S06.317A, S06.318A, S06.319A, S06.320A, S06.321A, S06.322A, S06.323A, S06.324A, S06.325A, S06.326A, S06.327A, S06.328A, S06.329A, S06.330A, S06.331A, S06.332A, S06.333A, S06.334A, S06.335A, S06.336A, S06.337A, S06.338A, S06.339A, S06.340A, S06.341A, S06.342A, S06.343A, S06.344A, S06.345A, S06.346A, S06.347A, S06.348A, S06.349A, S06.350A, S06.351A, S06.352A, S06.353A, S06.354A, S06.355A, S06.356A, S06.357A, S06.358A, S06.359A, S06.360A, S06.361A, S06.362A, S06.363A, S06.364A, S06.365A, S06.366A, S06.367A, S06.368A, S06.369A, S06.370A, S06.371A, S06.372A, S06.373A, S06.374A, S06.375A, S06.376A, S06.377A, S06.378A, S06.379A, S06.380A, S06.381A, S06.382A, S06.383A, S06.384A, S06.385A, S06.386A, S06.387A, S06.388A, S06.389A, S06.4X0A, S06.4X1A, S06.4X2A, S06.4X3A, S06.4X4A, S06.4X5A, S06.4X6A, S06.4X7A, S06.4X8A, S06.4X9A, S06.5X0A, S06.5X1A, S06.5X2A, S06.5X3A, S06.5X4A, S06.5X5A, S06.5X6A, S06.5X7A, S06.5X8A, S06.5X9A, S06.6X0A, S06.6X1A, S06.6X2A, S06.6X3A, S06.6X4A, S06.6X5A, S06.6X6A, S06.6X7A, S06.6X8A, S06.6X9A, S06.810A, S06.811A, S06.812A, S06.813A, S06.814A, S06.815A, S06.816A, S06.817A, S06.818A, S06.819A, S06.820A, S06.821A, S06.822A, S06.823A, S06.824A, S06.825A, S06.826A, S06.827A, S06.828A, S06.829A, S06.890A, S06.891A, S06.892A, S06.893A, S06.894A, S06.895A, S06.896A, S06.897A, S06.898A, S06.899A, S06.9X0A, S06.9X1A, S06.9X2A, S06.9X3A, S06.9X4A, S06.9X5A, S06.9X6A, S06.9X7A, S06.9X8A, S06.9X9A, OR Z51.89 as the principal DX Code then EXCLUDE. | Up to 6 months before the index date | At least 1 inpatient or 2 HOP or Carrier claims | CCW |

CCW – Chronic Conditions Warehouse

CPT – Current Procedural Terminology

DRG – Diagnosis Related Groups

GI – Gastrointestinal bleeding

HCPC – Healthcare Common Procedure Coding

HHA – Home Health Agency

HOP – Hospital Outpatient

ICD – International Classification of Disease

IP – Inpatient

SNF – Skilled Nursing Facility

USRDS – United State Renal Data System

VTE - Venous Thromboembolism

1 - For clinical covariates, ICD codes in any position were included. All listed codes are ICD diagnosis codes, unless otherwise noted.

**Table 3. Definitions of Outcomes**

| OUTCOME | ICD9^1^ | ICD10^1^ | SETTING |
| --- | --- | --- | --- |
| **Bleeding** |  |  |  |
| Major bleeding | Defined as a 1) a bleed associated with death^2^, 2) a critical site bleed (intracranial, intraocular, intra-articular, pericardial) associated with a hospitalization, or 3) a bleed associated with hospitalization and transfusion, as defined by Cunningham et al  ICD9 diagnosis codes for critical site bleeds^3^:  430, 431, 432.X, 363.6, 376.32, 377.42, 379.23, 719.1X, 729.92, 423.0, 568.81, 852.X, 853.X  ICD9 diagnosis codes for non-critical site bleeds:  285.1, 599.7, 599.71, 626.3,  626.9, 627.0, 627.1, 786.3,  786.39, 998.11, 998.12, 532.0X,  532.2X, 532.4X, 532.6X, 535.21, 562.13, 280.0, 459.0, 530.21, 530.82, 531.0X, 531.2X, 531.4X,  531.6X, 533.0X, 533.2X, 533.4X, 533.6X, 534.0X, 534.2X, 534.4X, 534.6X, 535.01, 535.11, 535.31,  535.41, 535.51, 535.61, 535.71,  537.83, 562.02, 562.03, 562.12,  569.3, 569.85, 569.86, 578.X,  596.7, 626.2, 626.5, 626.6,  626.7, 626.8, 664.5X, 666.0X,  666.1X, 666.2X, 784.7,  784.8, 860.2, 958.2  ICD9 procedure codes for blood transfusion:  99.00, 99.02, 99.03, 99.04  Additional codes to identify blood cell transfusion:  CPT 36430, c) HCPC: P9010, P9011, P9016, P9021, P9022, P9038-P9040, P9051, P9054, P9056–P9058 , d) Revenue Center Codes: 0380-0382, e) Additional Value Codes: 37, 38, 39 | Defined as a 1) a bleed associated with death^2^, 2) a critical site bleed (intracranial, intraocular, intra-articular, pericardial) associated with a hospitalization, or 3) a bleed associated with hospitalization and transfusion, as defined by Cunningham et al  ICD10 diagnosis codes for critical site bleeds^3^:  I60.X, I61.X, I62.X, H31.30X, H31.31X, H05.23X, H43.1X, H44.81X, H47.02X, M79.81, M25.0X, K66.1, I31.2, S06.4X, S06.5X, S06.6X  ICD10 diagnosis codes for non-critical site bleeds:  D50.0, D62, D69.8, D69.9, D78.01, D78.02, D78.21, D78.22, D78.31, D78.32, E36.01, E36.02, E89.810, E89.811, G97.31, G97.32, G97.51, G97.52, H59.111, H59.112, H59.113, H59.119, H59.121, H59.122, H59.123, H59.129, H59.311, H59.312, H59.313, H59.319, H59.321, H59.322, H59.323, H59.329, H95.21, H95.22, H95.41,H95.42, I97.410, I97.411, I97.418, I97.42, I97.610,  I97.611, I97.618, I97.620,  J95.61, J95.62, J95.830, J95.831, K20.81, K20.91, K21.01, K22.11, K25.0, K25.2, K25.4, K25.6, K26.0, K26.2, K26.4, K26.6, K27.0, K27.2, K27.4, K27.6, K28.0, K28.2, K28.4, K28.6, K29.01,K29.21, K29.31, K29.41, K29.51,K29.61, K29.71, K29.81, K29.91, K31.811, K31.82, K52.21, K57.01, K57.11, K57.13, K57.21, K57.31, K57.33, K57.41, K57.51, K57.53, K57.81, K57.91, K57.93, K62.5, K91.61, K91.62, K91.840,  K91.841, K92.0, K92.1, K92.2,  L76.01, L76.02, L76.21, L76.22,  M96.810, M96.811, M96.830,  M96.831, N92.0, N92.1,  N92.2, N92.3, N92.4, N93.0, N93.1, N93.8, N93.9, N99.510,  N99.520, N99.530, N99.61,  N99.62, N99.820, N99.821,  O67.0, O67.8, O67.9, O72.0,  O72.1 O72.2, R04.X, R31.0, R31.9, S27.1X, S27.2X,  T79.2X  ICD10 procedure codes for blood transfusion:  30230H0, 30230H1, 30230N0, 30230N1, 30230P0, 30230P1, 30233N0, 30233N1, 30233P0, 30233P1, 30233H0, 30233H1, 30240H0, 30240H1, 30240N0, 30240N1, 30240P0, 30240P1, 30243H0, 30243H1, 30243N0, 30243N1, 30243P0, 30243P1  Additional codes to identify blood cell transfusion:  CPT 36430, c) HCPC: P9010, P9011, P9016, P9021, P9022, P9038-P9040, P9051, P9054, P9056–P9058 , d) Revenue Center Codes: 0380-0382, e) Additional Value Codes: 37, 38, 39 | Hospital |
| Intracranial Bleed^3^ | 430, 431, 432.X, 852.X, 853.X | I60.X, I61.X, I62.X, S06.4X, S06.5X, S06.6X | Any setting |
| Gastrointestinal  Bleed | 532.0X, 532.2X, 532.4X, 532.6X, 535.21, 562.13, 530.21, 530.82, 531.0X, 531.2X, 531.4X, 531.6X, 533.0X, 533.2X, 533.4X, 533.6X, 534.0X, 534.2X, 534.4X,  534.6X, 535.01, 535.11, 535.31,  535.41, 535.51, 535.61, 535.71,  537.83, 562.02, 562.03, 562.12,  569.3, 569.85, 569.86, 578.X | K20.81, K20.91, K21.01, K22.11, K25.0, K25.2, K25.4, K25.6, K26.0, K26.2, K26.4, K26.6, K27.0, K27.2, K27.4, K27.6, K28.0, K28.2, K28.4, K28.6, K29.01, K29.21, K29.31, K29.41, K29.51,K29.61, K29.71, K29.81, K29.91, K31.811, K31.82, K52.21, K57.01, K57.11, K57.13, K57.21, K57.31, K57.33, K57.41, K57.51, K57.53, K57.81, K57.91, K57.93, K62.5, K92.0, K92.1, K92.2 | Hospital |
| Clinically relevant, non-major bleed | Critical site bleeding codes  Non-critical site bleeding codes  (without blood transfusion) | Critical site bleeding codes  Non-critical site bleeding codes  (without blood transfusion) | Non-hospital setting  Hospital |
| **Recurrent VTE^4^** | 415.11, 415.13, 415.19, 451.11,  451.19, 451.2, 451.81, 451.83,  451.84, 451.89, 451.9, 453.2,  453.3, 453.4, 453.41, 453.42,  453.82, 453.83, 453.84, 453.85,  453.86, 453.87, 453.89, 453.9,  671.3, 671.4, 671.5, 673.2,  673.8 | I26.02, I26.09, I26.92, I26.93  I26.94, I26.99, I80.10, I80.11,  I80.12, I80.13, I80.201, I80.202,  I80.203, I80.209, I80.211, I80.212, I80.213, I80.219, I80.221, I80.222, I80.223, I80.229, I80.231, I80.232,  I80.233, I80.239, I80.241, I80.242, I80.243, I80.249, I80.251, I80.252, I80.253, I80.259, I80.291, I80.292,  I80.293, I80.299, I82.210, I82.220, I82.290, I82.3,  I82.401, I82.402, I82.403, I82.409, I82.411, I82.412, I82.413, I82.419, I82.421, I82.422, I82.423, I82.429, I82.431, I82.432, I82.433, I82.439, I82.441, I82.442, I82.443, I82.449, I82.451, I82.452, I82.453, I82.459, I82.461, I82.462, I82.463, I82.469, I82.491, I82.492, I82.493, I82.499, I82.4Y1, I82.4Y2, I82.4Y3, I82.4Y9, I82.4Z1, I82.4Z2, I82.4Z3, I82.4Z9, I82.601, I82.602, I82.603, I82.609, I82.621, I82.622, I82.623, I82.629, I82.890, I82.90, I82.A11, I82.A12, I82.A13, I82.A19, I82.B11, I82.B12, I82.B13, I82.B19, I82.C11, I82.C12, I82.C13, I82.C19, O22.30, O22.31, O22.32, O22.33,  O88.211, O88.212, O88.213, O88.219, O88.22, O88.23, O88.811, O88.812, O88.813, O88.819, O88.82, O88.83, T82.818A, T82.868A | Hospital |

CPT – Current Procedural Terminology

ICD – International Classification of Disease

VTE – Venous thromboembolism

Footnotes:

1 - All ICD codes must be in the primary position.

2 – Death during a hospitalization or within a week of hospital discharge from a bleeding-associated hospitalization.

3 - We excluded only those traumatic intracranial bleeds that were associated with skull fractures.

4 – Diagnosis of recurrent VTE must be greater than 21 days after index prescription for anticoagulant.

**Table 4. Cohort Creation**

| **Apixaban** | | **Criteria** | **Warfarin** | |
| --- | --- | --- | --- | --- |
| Remaining patients | Excluded patients |  | Excluded patients | Remaining patients |
| 48,461 |  | Patients who had at least one fill for the index drug during the index period (January 1, 2014 – June 30, 2018) (the first date of fill is index date) |  | 146,558 |
| 48,439 | 22 | Patients who were ≥ 18 years old at index date | 93 | 146,465 |
| 24,213 | 24,226 | Patients who had continuous enrollment in Medicare parts A and B during the six months prior to index date | 75,057 | 71,408 |
| 22,263 | 1,950 | Patients who had continuous enrollment in Medicare part D for the six months prior to index date | 6,744 | 64,664 |
| 6,383 | 15,880 | Patients who had an acute VTE diagnosis between 30 days before and 7 days after index date | 45,926 | 18,738 |
| 5,109 | 1,274 | Excluded due to having ≥  60 days cumulative use of anticoagulant (DOAC, warfarin, heparin, low-molecular weight heparin) from six months to one day before index date | 3,400 | 15,338 |
| 4,690 | 419 | Excluded due to use of any of DOACs or warfarin from one month to one day before index date | 418 | 14,920 |
| 3,650 | 1,040 | Excluded due to having at least one inpatient or two outpatient or carrier file diagnoses of atrial fibrillation during the six months prior to the index date | 3,033 | 11,887 |
| 3,631 | 19 | Excluded due to admission to hospice during six months prior to index date | 52 | 11,835 |
| 3,179 | 452 | Excluded because the patient filled the other index drug at any time from January 1, 2014, to index date (this step is added to create mutually exclusive cohorts) | 100 | 11,735 |
| 2,401 | 778 | Excluded because the patient had a kidney transplant and was not on dialysis up to index date | 2,162 | 9,573 |
| 2,302 | 99 | Excluded because the patient was not on dialysis for the six months prior to index date | 310 | 9,263 |
| **2,302** |  | **Final cohort** |  | **9,263** |

DOAC – apixaban, edoxaban, dabigatran, rivaroxaban

**Figure 2. Change in Apixaban and Warfarin Use by Year**


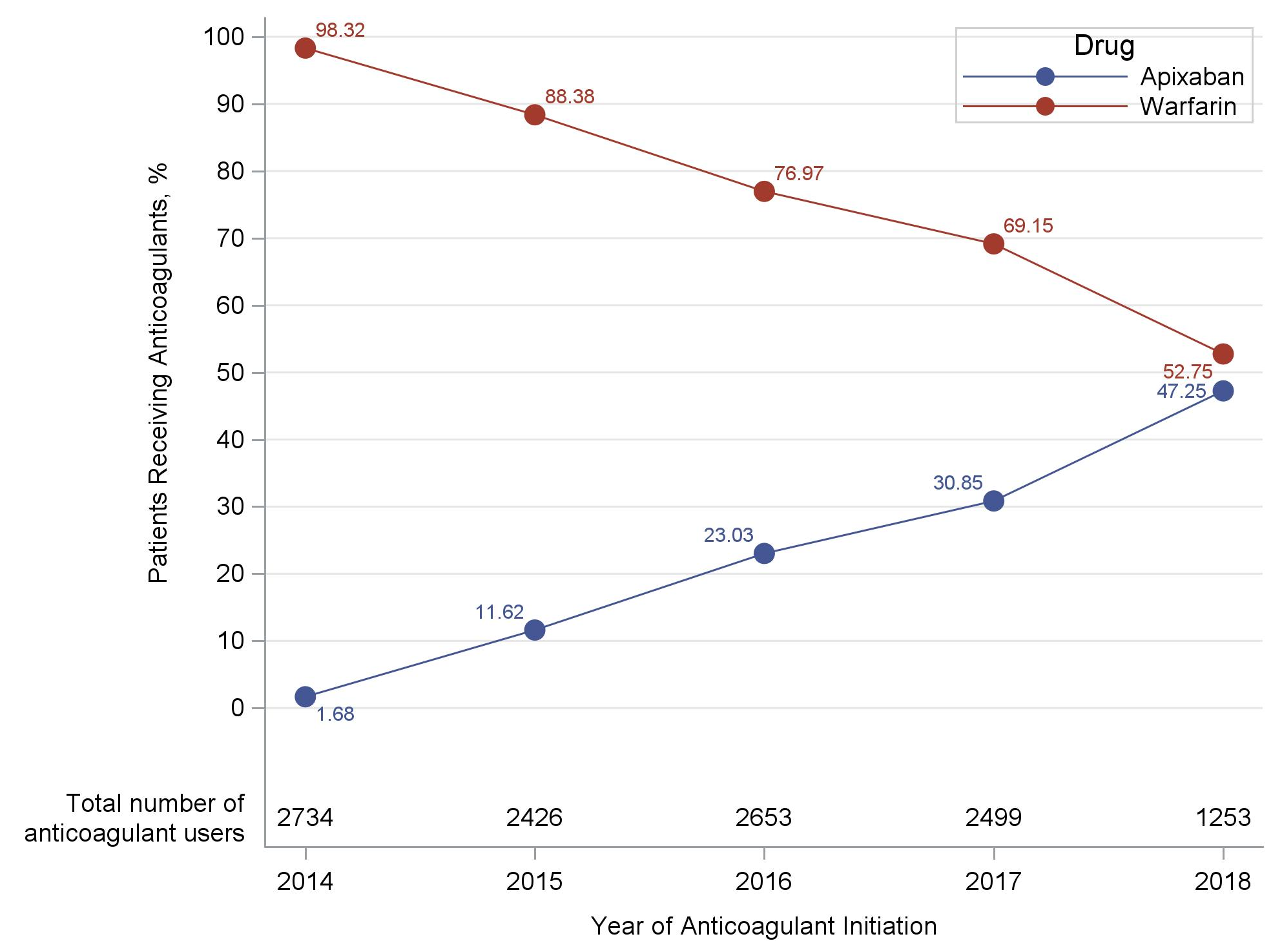


**Table 5. Mean Follow-Up Days for Outcomes**

|  | **Total** | **Apixaban** | **Warfarin** |
| --- | --- | --- | --- |
| **Total Major bleeding**  Mean follow-up days (standard deviation) | 151.8 (56.9) | 153.4 (56.2) | 151.4 (57.1) |
| **Fatal major bleeding** | 162.8 (47.1) | 162.3 (47.8) | 162.9 (46.9) |
| **Non-fatal major bleeding** | 152.2 (56.4) | 153.7 (55.8) | 151.8 (56.6) |
| **Total clinically relevant non-major bleeding** | 145.3 (60.8) | 147.8 (59.5) | 144.7 (61.1) |
| **Intracranial bleeding** | 161.3 (48.5) | 161.3 (48.9) | 161.3 (48.4) |
| **GI bleeding** | 153.9 (55.3) | 154.7 (55.0) | 153.7 (55.3) |
| **Recurrent VTE** | 157.7 (50.8) | 157.1 (51.3) | 157.8 (50.6) |
| **All-cause mortality** | 163.2 (46.4) | 162.6 (47.3) | 163.4 (46.1) |

For intention-to-treat analysis with 6-month follow-up

GI – Gastrointestinal

VTE – Venous Thromboembolism

**Table 6. Counts of blood transfusion episodes per patient**

|  | **Total** | **Apixaban** | **Warfarin** | **Poisson regression results*** | | |
| --- | --- | --- | --- | --- | --- | --- |
|  |  |  |  | Poisson coefficient | | P-value |
|  |  |  |  | Apixaban | Warfarin |  |
| **Intention-to-treat (6 months)** |  |  |  |  |  |  |
| Mean (SD) | 0.37 (0.91) | 0.35 (1.0) | 0.37 (0.89) | 0.12 | Ref. | 0.08 |
| **Intention-to-treat (3 months)** |  |  |  |  |  |  |
| Mean (SD) | 0.22 (0.61) | 0.21 (0.65) | 0.22 (0.59) | 0.09 | Ref. | 0.23 |
| **Intention-to-treat (1 month)** |  |  |  |  |  |  |
| Mean (SD) | 1.0 (0.34) | 1.0 (0.35) | 1.0 (0.34) | 0.12 | Ref. | 0.22 |
| **As-treated (6 months)** |  |  |  |  |  |  |
| Mean (SD) | 0.19 (0.59) | 0.16 (0.58) | 0.21 (0.59) | -0.09 | Ref. | 0.27 |

* - Adjusted for Inverse Probability Treatment Weighing and index year

SD – Standard Deviation

**Table 7. Bleeding and Thrombotic Outcomes at 3 months, Intention-To-Treat Analysis**

|  | **Total** | **Apixaban** | **Warfarin** |
| --- | --- | --- | --- |
| Number of patients, N | 11,565 | 2,302 | 9,263 |
| **Total Major bleeding *** |  |  |  |
| Patients, N (%) | 1,003 (8.7) | 156 (6.8) | 847 (9.1) |
| IPTW and index year adjusted, HR (95%CI) |  | **0.78 (0.65-0.94)** | Ref. |
| **Fatal major bleeding** |  |  |  |
| Patients, N (%) | 187 (1.6) | 27 (1.2) | 160 (1.7) |
| IPTW and index year adjusted, HR (95%CI) |  | 0.66 (0.43-1.01) | Ref. |
| **Non-fatal major bleeding** |  |  |  |
| Patients, N (%) | 828 (7.2) | 131 (5.7) | 697 (7.5) |
| IPTW and index year adjusted, HR (95%CI) |  | **0.81(0.66-0.99)** | Ref. |
| **Total clinically relevant non-major bleeding** |  |  |  |
| Patients, N (%) | 1,397 (12.1) | 234 (10.2) | 1,163 (12.6) |
| IPTW and index year adjusted, HR (95%CI) |  | **0.78 (0.68-0.91)** | Ref. |
| **Intracranial bleeding** |  |  |  |
| Patients, N (%) | 188 (1.6) | 29 (1.3) | 159 (1.7) |
| IPTW and index year adjusted, HR (95%CI) |  | 0.69 (0.45-1.06) | Ref. |
| **GI bleeding** |  |  |  |
| Patients, N (%) | 770 (6.7) | 127 (5.5) | 643 (6.9) |
| IPTW and index year adjusted, HR (95%CI) |  | **0.74 (0.61-0.91)** | Ref. |
| **Recurrent VTE** |  |  |  |
| Patients, N (%) | 407 (3.5) | 77 (3.3) | 330 (3.6) |
| IPTW and index year adjusted, HR (95%CI) |  | **0.76 (0.59-0.98)** | Ref. |
| **All-cause mortality (censored at 91 days)** |  |  |  |
| Patients, N (%) | 709 (6.1) | 138 (6.0) | 571 (6.2) |
| IPTW and index year adjusted, HR (95%CI) |  | 1.01 (0.83-1.23) | Ref. |

* - Total major bleeding is slightly less than fatal major bleeding and non-fatal major bleeding combined because some patients had a non-fatal major bleeding hospitalization followed by a fatal major bleeding hospitalization.

Hazard rates were generated using intent-to-treat principle, defined by the medication first prescribed. We utilized a propensity score by modeling the probability of treatment with apixaban rather than warfarin for each cohort member using a logistic regression model including all available clinical covariates. We then weighted the individuals in the cohort using inverse probability of treatment weights (IPTW) after truncating the weights at the 1^st^ and 99^th^ percentile.

CI – Confidence Interval

GI – Gastrointestinal

HR – Hazard Ratio

IPTW – Inverse Probability of Treatment Weighting

VTE – Venous Thromboembolism

**Table 8. Bleeding and Thrombotic Outcomes at 1 month, Intention-To-Treat Analysis**

|  | **Total** | **Apixaban** | **Warfarin** |
| --- | --- | --- | --- |
| Number of patients, N | 11,565 | 2,302 | 9,263 |
| **Total Major bleeding *** |  |  |  |
| Patients, N (%) | 535 (4.6) | 86 (3.7) | 449 (4.9) |
| IPTW and index year adjusted, HR (95%CI) |  | 0.82 (0.64-1.06) | Ref. |
| **Fatal major bleeding** |  |  |  |
| Patients, N (%) | 90 (0.8) | 10 (0.4) | 80 (0.9) |
| IPTW and index year adjusted, HR (95%CI) |  | 0.53 (0.27-1.04) | Ref. |
| **Non-fatal major bleeding** |  |  |  |
| Patients, N (%) | 447 (3.9) | 76 (3.3) | 371 (4.0) |
| IPTW and index year adjusted, HR (95%CI) |  | 0.89 (0.68-1.17) | Ref. |
| **Total clinically relevant non-major bleeding** |  |  |  |
| Patients, N (%) | 708 (6.1) | 116 (5.0) | 592 (6.4) |
| IPTW and index year adjusted, HR (95%CI) |  | **0.76 (0.62-0.94)** | Ref. |
| **Intracranial bleeding** |  |  |  |
| Patients, N (%) | 91 (0.8) | 18 (0.8) | 73 (0.8) |
| IPTW and index year adjusted, HR (95%CI) |  | 0.99 (0.56-1.74) | Ref. |
| **GI bleeding** |  |  |  |
| Patients, N (%) | 410 (3.6) | 71 (3.1) | 339 (3.7) |
| IPTW and index year adjusted, HR (95%CI) |  | 0.81 (0.61-1.06) | Ref. |
| **Recurrent VTE** |  |  |  |
| Patients, N (%) | 98 (0.9) | 16 (0.7) | 82 (0.9) |
| IPTW and index year adjusted, HR (95%CI) |  | 0.67 (0.39-1.17) | Ref. |
| **All-cause mortality** |  |  |  |
| Patients, N (%) | 267 (2.3) | 52 (2.3) | 215 (2.3) |
| IPTW and index year adjusted, HR (95%CI) |  | 0.98 (0.71-1.35) | Ref. |

* - Total major bleeding is slightly less than fatal major bleeding and non-fatal major bleeding combined because some patients had a non-fatal major bleeding hospitalization followed by a fatal major bleeding hospitalization.

Hazard rates were generated using intent-to-treat principle, defined by the medication first prescribed. We utilized a propensity score by modeling the probability of treatment with apixaban rather than warfarin for each cohort member using a logistic regression model including all available clinical covariates. We then weighted the individuals in the cohort using inverse probability of treatment weights (IPTW) after truncating the weights at the 1^st^ and 99^th^ percentile.

CI – Confidence Interval

GI – Gastrointestinal

HR – Hazard Ratio

IPTW – Inverse Probability of Treatment Weighting

VTE – Venous Thromboembolism

**Table 9. Bleeding and Thrombotic Outcomes at 6 months, As-Treated Analysis**

|  | **Total** | **Apixaban** | **Warfarin** |
| --- | --- | --- | --- |
| Number of patients, N | 11,565 | 2,302 | 9,263 |
| **Total Major bleeding *** |  |  |  |
| Patients, N | 973 (8.4) | 140 (6.1) | 833 (9.0) |
| IPTW and index year adjusted, HR (95%CI) |  | **0.82 (0.67-0.99)** | Ref. |
| **Fatal major bleeding** |  |  |  |
| Patients, N | 160 (1.4) | 24 (1.0) | 136 (1.5) |
| IPTW and index year adjusted, HR (95%CI) |  | 0.81 (0.51-1.29) | Ref. |
| **Non-fatal major bleeding** |  |  |  |
| Patients, N | 820 (7.1) | 118 (5.1) | 702 (7.6) |
| IPTW and index year adjusted, HR (95%CI) |  | 0.83 (0.67-1.03) | Ref. |
| **Total clinically relevant non-major bleeding** |  |  |  |
| Patients, N | 1,350 (11.7) | 194 (8.4) | 1156 (12.5) |
| IPTW and index year adjusted, HR (95%CI) |  | **0.76 (0.65-0.89)** | Ref. |
| **Intracranial bleeding** |  |  |  |
| Patients, N | 175 (1.5) | 27 (1.2) | 148 (1.6) |
| IPTW and index year adjusted, HR (95%CI) |  | 0.79 (0.51-1.23) | Ref. |
| **GI bleeding** |  |  |  |
| Patients, N | 752 (6.5) | 115 (5.0) | 637 (6.9) |
| IPTW and index year adjusted, HR (95%CI) |  | **0.81 (0.65-0.999)** | Ref. |
| **Recurrent VTE** |  |  |  |
| Patients, N | 413 (3.6) | 70 (3.0) | 343 (3.7) |
| IPTW and index year adjusted, HR (95%CI) |  | 0.77 (0.59-1.01) | Ref. |
| **All-cause mortality** |  |  |  |
| Patients, N | 491 (4.3) | 94 (4.1) | 397 (4.3) |
| IPTW and index year adjusted, HR (95%CI) |  | 1.14 (0.89-1.45) | Ref. |

* - Total major bleeding is slightly less than fatal major bleeding and non-fatal major bleeding combined because some patients had a non-fatal major bleeding hospitalization followed by a fatal major bleeding hospitalization.

Hazard rates were generated using as-treated principle, with censoring for medication discontinuation or switching. We utilized a propensity score by modeling the probability of treatment with apixaban rather than warfarin for each cohort member using a logistic regression model including all available clinical covariates. We then weighted the individuals in the cohort using inverse probability of treatment weights (IPTW) after truncating the weights at the 1^st^ and 99^th^ percentile.

CI – Confidence Interval

GI – Gastrointestinal

HR – Hazard Ratio

IPTW – Inverse Probability of Treatment Weighting

VTE – Venous Thromboembolism

**Table 10. Comparison of Patient Demographics, Comorbidities, and Medication Exposures Between 2.5mg and 5mg Apixaban Subgroups**

| **Characteristic** | **Apixaban 2.5 mg** | **Apixaban 5 mg** | **P-value** |
| --- | --- | --- | --- |
| Number | 933 | 1,150 |  |
| Age at index date, Years |  |  |  |
| Mean (±SD) | 62.2 (15.4) | 57.9 (14.7) | **<0.0001** |
| Gender (%) |  |  |  |
| Male | 378 (40.5) | 557 (48.4) |  |
| Female | 555 (59.5) | 593 (51.6) | **0.0003** |
| Race (%) |  |  |  |
| White | 446 (47.8) | 514 (44.7) |  |
| Black / African American | 454 (48.7) | 590 (51.3) |  |
| Other | 33 (3.5) | 46 (4.0) | 0.35 |
| Ethnicity (%) |  |  |  |
| Hispanic | 141 (15.1) | 142 (12.4) |  |
| Non-Hispanic | 791 (84.8) | 1,006 (87.5) |  |
| Unknown | 1 (0.1) | 2 (0.2) | 0.17 |
| Dual-eligible (%) | 393 (42.1) | 500 (43.5) | 0.53 |
| Year of index medication fill (%) |  |  |  |
| 2014 | 18 (1.9) | 22 (1.9) |  |
| 2015 | 99 (10.6) | 166 (14.4) |  |
| 2016 | 240 (25.7) | 309 (26.9) |  |
| 2017 | 314 (33.7) | 379 (33.0) |  |
| 2018 | 262 (28.1) | 274 (23.8) | **0.04** |
| Time on dialysis (years) (%) |  |  |  |
| <1 | 65 (7.0) | 82 (7.1) |  |
| 1-2 | 122 (13.1) | 175 (15.2) |  |
| 2-3 | 103 (11.0) | 128 (11.1) |  |
| ≥3 | 639 (68.5) | 758 (65.9) |  |
| Unknown | 4 (0.4) | 7 (0.6) | 0.64 |
| Dialysis modality at index date |  |  |  |
| Hemodialysis | 886 (95.0) | 1,092 (95.0) |  |
| Peritoneal dialysis | 47 (5.0) | 58 (5.0) | 0.99 |
| Anemia (%) | 930 (99.7) | 1,147 (99.7) | 0.80 |
| Diabetes (%) | 633 (67.9) | 802 (69.7) | 0.35 |
| Hypertension (%) | 914 (98.0) | 1,115 (97.0) | 0.15 |
| Ischemic Heart Disease (%) | 531 (56.9) | 617 (53.7) | 0.14 |
| Obesity (%) | 505 (54.1) | 723 (62.9) | **<0.0001** |
| Peptic ulcer disease (%) | 43 (4.6) | 55 (4.8) | 0.85 |
| Pulmonary hypertension (%) | 82 (8.8) | 95 (8.3) | 0.67 |
| Smoker (%) | 273 (29.3) | 383 (33.3) | **0.048** |
| Prior VTE (%) | 413 (44.3) | 445 (38.7) | **0.01** |
| Prior GI bleeding (%) | 130 (13.9) | 157 (13.7) | 0.85 |
| Peripheral arterial disease (%) | 346 (37.1) | 402 (35.0) | 0.31 |
| Liver disease (except viral hepatitis) | 82 (8.8) | 96 (8.4) | 0.72 |
| Active malignancy (%) | 102 (10.9) | 114 (9.9) | 0.45 |
| Prescence of central catheter (%) | 519 (55.6) | 631 (54.9) | 0.73 |
| Recent trauma^1^ (%) | 170 (18.2) | 196 (17.0) | 0.48 |
| Recent surgery^2^ (%) | 308 (33.0) | 335 (29.1) | 0.06 |
| Heart failure (%) | 367 (39.3) | 488 (42.4) | 0.15 |
| Prior major bleeding | 105 (11.3) | 104 (9.0) | 0.09 |
| Stroke | 168 (18.0) | 173 (15.0) | 0.07 |
| Antiplatelet^3^ (other than aspirin) (%) | 209 (22.4) | 258 (22.4) | 0.99 |
| Vasodilators^4^ (%) | 19 (2.0) | 16 (1.4) | 0.25 |
| Cox2 selective NSAIDs (%) | 7 (0.8) | 6 (0.5) | 0.51 |
| Cox2 nonselective NSAIDs (%) | 85 (9.1) | 101 (8.8) | 0.79 |
| PPI (%) | 415 (44.5) | 517 (45.0) | 0.83 |
| Antidepressants^5^ (%) | 221 (23.7) | 359 (31.2) | **0.0001** |
| H2 blockers (%) | 133 (14.3) | 146 (12.7) | 0.30 |
| Continuous use of oral corticosteroids (<1 week) | 87 (9.3) | 107 (9.3) | 0.99 |
| Continuous use of Oral Corticosteroids (≥1 week) | 119 (12.8) | 150 (13.0) | 0.85 |
| Erythropoiesis stimulating agents | 860 (92.2) | 1,016 (88.4) | **0.004** |
| Aspirin (%)* | 7 (0.8) | 5 (0.4) | 0.34 |

COX – Cyclooxygenase

mg – milligrams

NSAIDs – Nonsteroidal Anti-Inflammatory Drugs

PPI – Proton Pump Inhibitor

TIA – Transient Ischemic Attack

VTE – Venous Thromboembolism

*Prescription use only; USRDS does not include over-the-counter medications; aspirin was not included as a covariate in modeling of outcomes

1 – Recent trauma defined as up to 30 days before index date (eTable 2)

2 – Recent surgery defined as up to 60 days before index date (eTable 2)

3 – Clopidogrel, Prasugrel, Ticagrelor, Ticlodipine

4 – Cilostazol, Dipyramidole

5 – Citalopram, Escitalopram, Fluoxetine, Fluvoxamine, Paroxetine, Sertraline, Nefazodone, Trazodone, Amitriptyline, Clomipramine

**Table 11. Comparison of Outcomes Between Apixaban Dosing Subgroups – 5 mg Versus 2.5mg**

|  | **Apixaban 2.5 mg Subgroup** | **Apixaban 5 mg Subgroup** |
| --- | --- | --- |
| Number of patients, N | 933 | 1,150 |
| Total Major bleeding, HR (95% CI) | 0.87 (0.65-1.16) | Ref. |
| Fatal major bleeding | 1.14 (0.59-2.22) | Ref. |
| Non-fatal major bleeding | 0.81 (0.58-1.12) | Ref. |
| Total clinically relevant non-major bleeding | 0.98 (0.78-1.25) | Ref. |
| Intracranial bleeding | 0.61 (0.28-1.34) | Ref. |
| GI bleeding | **0.69 (0.50-0.94)** | Ref. |
| Recurrent VTE | 1.07 (0.75-1.53) | Ref. |
| All-cause mortality | 1.06 (0.80-1.42) | Ref. |

Using Intention-to-treat, with six-month follow-up and direct adjustment for covariates

CI – Confidence Interval

GI – Gastrointestinal

HR – Hazard Ratio

mg – Milligrams

VTE – Venous Thromboembolism

**Table 12. Comparison of Outcomes Between Apixaban 2.5mg Subgroup and Warfarin Cohort**

|  | **Apixaban 2.5 mg Subgroup** | **Warfarin Cohort** |
| --- | --- | --- |
| Number of patients, N | 933 | 9,263 |
| Total Major bleeding, HR (95% CI) | **0.68 (0.54-0.85)** | Ref. |
| Fatal major bleeding | 0.75 (0.48-1.18) | Ref. |
| Non-fatal major bleeding | **0.67 (0.52-0.87)** | Ref. |
| Total clinically relevant non-major bleeding | **0.78 (0.65-0.93)** | Ref. |
| Intracranial bleeding | **0.48 (0.27-0.84)** | Ref. |
| GI bleeding | **0.61 (0.47-0.79)** | Ref. |
| Recurrent VTE | 0.81 (0.62-1.06) | Ref. |
| All-cause mortality | 1.08 (0.87-1.34) | Ref. |

Using Intention-to-treat, with six-month follow-up and direct adjustment for covariates

CI – Confidence Interval

GI – Gastrointestinal

HR – Hazard Ratio

mg – Milligrams

VTE – Venous Thromboembolism

**Table 13. Comparison of Outcomes Between Apixaban 5mg Subgroup and Warfarin Cohort**

|  | **Apixaban 5 mg Subgroup** | **Warfarin Cohort** |
| --- | --- | --- |
| Number of patients, N | 1,150 | 9,263 |
| Total Major bleeding, HR (95% CI) | **0.76 (0.62-0.93)** | Ref. |
| Fatal major bleeding | **0.60 (0.36-0.98)** | Ref. |
| Non-fatal major bleeding | 0.82 (0.66-1.02) | Ref. |
| Total clinically relevant non-major bleeding | **0.77 (0.65-0.90)** | Ref. |
| Intracranial bleeding | 0.72 (0.45-1.16) | Ref. |
| GI bleeding | 0.87 (0.71-1.08) | Ref. |
| Recurrent VTE | **0.76 (0.58-0.98)** | Ref. |
| All-cause mortality | 0.98 (0.79-1.21) | Ref. |

Using Intention-to-treat, with six-month follow-up and direct adjustment for covariates

CI – Confidence Interval

GI – Gastrointestinal

HR – Hazard Ratio

mg – Milligrams

VTE – Venous Thromboembolism

**Table 14. Comparison of Hazard Ratios for IPTW Modeling and Direct Covariate Adjustment for Outcomes in Intention-to-Treat Analysis with Six Month Follow-Up**

| **Outcomes** | **IPTW and index year-adjusted, HR (95% CI)** | **Direct adjusted model for all covariates, HR (95% CI)** |
| --- | --- | --- |
| Total major bleeding | **0.81 (0.70-0.94)** | **0.77 (0.66-0.90)** |
| Fatal major bleeding | 0.71 (0.51-1.00) | **0.66 (0.47-0.93)** |
| Non-fatal major bleeding | 0.85 (0.72-1.004) | **0.81 (0.69-0.96)** |
| Clinically relevant non-major bleeding | **0.84 (0.74-0.94)** | **0.80 (0.71-0.91)** |
| Intracranial bleeding | **0.69 (0.48-0.98)** | **0.63 (0.44-0.91)** |
| GI bleeding | **0.82 (0.69-0.96)** | **0.78 (0.66-0.93)** |
| Recurrent VTE | 0.83 (0.69-1.002) | 0.83 (0.69-1.01) |
| All-cause mortality | 1.06 (0.91-1.24) | 1.03 (0.86-1.17) |

CI – Confidence Interval

GI – Gastrointestinal

HR – Hazard Ratio

IPTW – Inverse Probability of Treatment Weighting

VTE – Venous Thromboembolism
